# A generic workflow for Single Locus Sequence Typing (SLST) design and subspecies characterization of microbiota

**DOI:** 10.1038/s41598-019-56065-y

**Published:** 2019-12-27

**Authors:** Thomas H. A. Ederveen, Jos P. H. Smits, Karima Hajo, Saskia van Schalkwijk, Tessa A. Kouwenhoven, Sabina Lukovac, Michiel Wels, Ellen H. van den Bogaard, Joost Schalkwijk, Jos Boekhorst, Patrick L. J. M. Zeeuwen, Sacha A. F. T. van Hijum

**Affiliations:** 1 Center for Molecular and Biomolecular Informatics (CMBI), Radboud Institute for Molecular Life Sciences (RIMLS), Radboud University Medical Center (Radboudumc), Nijmegen, The Netherlands; 2 Department of Dermatology, RIMLS, Radboudumc, Nijmegen, The Netherlands; 3 NIZO, Ede, The Netherlands

**Keywords:** Classification and taxonomy, Phylogeny, Next-generation sequencing, Microbiome, Bacterial genes

## Abstract

We present TaxPhlAn, a new method and bioinformatics pipeline for design and analysis of single-locus sequence typing (SLST) markers to type and profile bacteria beyond the species-level in a complex microbial community background. TaxPhlAn can be applied to any group of phylogenetically-related bacteria, provided reference genomes are available. As TaxPhlAn requires the SLST targets identified to fit the phylogenetic pattern as determined through comprehensive evolutionary reconstruction of input genomes, TaxPhlAn allows for the identification and phylogenetic inference of new biodiversity. Here, we present a clinically relevant case study of high-resolution *Staphylococcus* profiling on skin of atopic dermatitis (AD) patients. We demonstrate that SLST enables profiling of cutaneous *Staphylococcus* members at (sub)species level and provides higher resolution than current 16S-based techniques. With the higher discriminative ability provided by our approach, we further show that the presence of *Staphylococcus capitis* on the skin together with *Staphylococcus aureus* associates with AD disease.

## Introduction

Consortia of bacteria are found in many niches and there is increasing evidence of bacterial involvement in health and disease^1^. Bacterial diversity is considerable, and the current challenge lies in determining which bacteria and corresponding functionalities are relevant in a given ecological niche. In order to perform follow-up experiments in *in vitro* or animal models with candidate bacteria that are assumed to be important for a given niche, resolution down to the strain level is desirable^2^. Traditionally, bacterial occurrence is determined through culture-based methods, with subsequent isolate identification by a plethora of available phylo- and genotyping methods: chemotaxonomy^3^, DNA fingerprinting^4,5^, (quantitative) PCR^6^, mass spectrometry^7^ and genome sequencing^8^. One well-established genotyping method for bacterial strains is multi-locus sequence typing (MLST)^9^, which is based on sequence variety in a number of marker core genes revealed by qPCR and Sanger sequencing. However, most of the aforementioned techniques have limited resolution, and placing a novel genotype in its correct phylogenetic context is usually difficult. Notably, single strain full genome sequencing does not suffer from these drawbacks, but is more costly, labor intensive, and again, not easily compatible with high throughput applications^10^. Furthermore, DNA fingerprinting, MLST and genome sequencing do not allow for the measurement of bacterial abundances, and not all bacteria can be cultivated efficiently. Currently, sequencing-based culture-free analysis of complex microbial consortia as a whole can be performed with approaches such as 16S rRNA marker gene sequencing (16S metataxonomics) or shotgun metagenomics^11^. 16S metataxonomics focuses on 16S rRNA genes, which are universally present in all bacteria, is relatively cheap and data analysis is straightforward^12^. Depending on the primer set, 16S allows for confidently profiling most bacteria down to the genus level^13,14^. Metagenomics sequences in principle all free DNA present in a sample, allowing the classification of sequences at high taxonomical resolution as well as determining functionality present in a microbiome^15,16^. Recently, computational analysis methods were adopted for strain-level classification of metagenomics sequencing data, such as ConStrains^17^, PanPhlAn^18^ and StrainPhlAn^19^. However, obtaining sufficient biomaterial for metagenomics as well as generating and analyzing the datasets requires significant resources. Furthermore, aforementioned strain-level classification methods have difficulty with confidently detecting bacterial entities under a relative abundance level threshold of approximately 1% in a metagenomics sample^17^. Marker gene sequencing approaches perfectly allow for profiling bacteria that are below 0.1% relative abundance (i.e. 1 in 10,000 reads). Recently, single locus sequence typing (SLST) has been described for determining down-to strain level identification of *Propionibacterium acnes* human isolates by Scholz *et al*.^20,21^. Other applications of SLST have been reported for *Lactobacillus plantarum* strain tracking in human gut^22^ and industrial biofilms^23^, and for *Staphylococcus (aureus)* profiling on skin of atopic dermatitis patients during therapeutic intervention with coal tar^24^.

**Figure 1:**
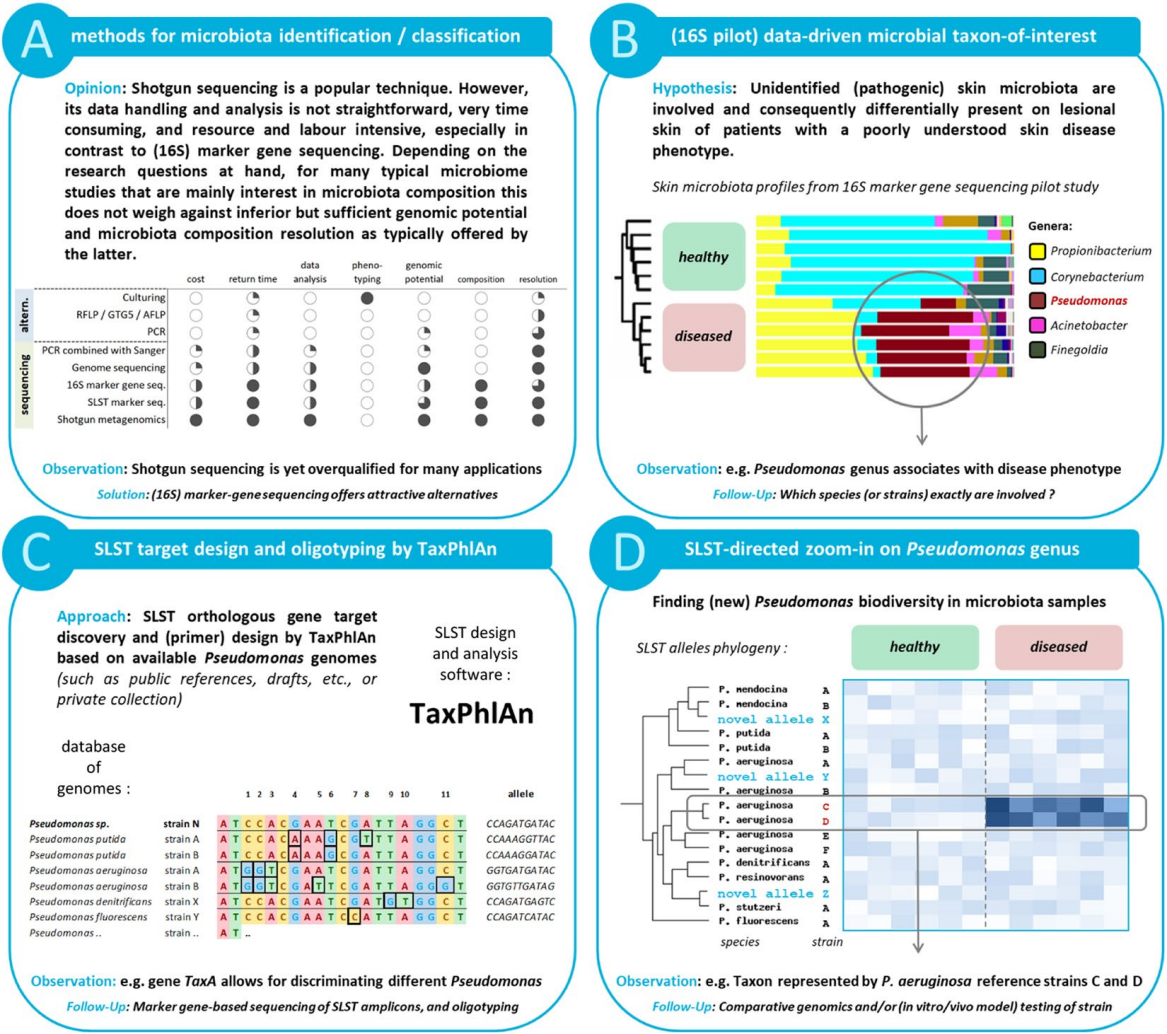
SLST allows high-resolution discrimination of different bacterial phylotypes, (sub)species and strains. (**A**) Overview of current sequencing-based and alternative methods for microbiota identification and classification, including their (dis)advantages. Table legend as follows: estimation and indication of *costs*, *return time* and *data analysis complexity;* methods that allow for *accurate phenotyping*; amount of information retrieved with regard to *genomic (functional) potential*, taxonomic *composition* and *resolution*. The symbols represent open versus closed pie charts, meaning: low vs. high, fast vs. slow, simple vs. complex, etc. **(B**) Hypothetical dataset with 16S sequencing microbiota data of “healthy” and “diseased” subjects who suffer from an exemplary skin disease. The colored bars represent skin microbiota composition, and is clustered on microbiota profiles. In this hypothetical example, the bacterium *Pseudomonas* associates with skin disease state of the volunteers. **(C**) *Pseudomonas* genomes are collected and analyzed with the bioinformatics tool TaxPhlAn in order to search for candidate SLST targets. The goal of TaxPhlAn is to find genetic regions that allow for as perfect as possible discrimination of *Pseudomonas* biodiversity. Shown is a hypothetical SLST region (aligned DNA sequences) that allows for discriminating different *Pseudomonas* (sub)species. (**D**) Hypothetical example of how the SLST target as identified in (**C**) could allow for high-resolution *Pseudomonas* typing, and thereby to determine the (sub)species or strain-level bacteria that associate with disease. Panel represents an heatmap with data of identified *Pseudomonas* biodiversity, and their relative abundance as measured for each sample. Available (clinical) isolates of these bacteria can be subsequent candidates for follow-up studies.

Until now, an automated bioinformatics pipeline to devise sequence-based SLST screening tools for specific microbes in complex microbial communities is lacking, and currently requires mostly manual searches with a lot of hands-on time. We here present TaxPhlAn, which stands for SLST-based **Tax**onomy **Ph**y**l**ogenetic **An**alysis: a method and workflow to create and use single locus marker sequences of orthologous genes to profile specific bacterial taxa at and beyond the species level (as illustrated in Fig. 1, based on a toy example with *Pseudomonas*). The TaxPhlAn bioinformatics pipeline finds SLST marker genes based on a set of reference genomes provided by the user in module A of the pipeline (Suppl. Fig. S1). Candidate SLST regions selected by the TaxPhlAn pipeline balance a trade-off between sequence conservation, which is important for PCR primer design and identification, and sequence variation within single-copy genes shared among all genomes-of-interest to allow for a certain degree of discrimination between genomes. SLST markers are designed to follow the actual phylogeny of the considered strains, and additionally scored based on characteristics such as discriminative resolution, marker length (i.e. amplicon size) and level of conservation of the marker in the taxon-of-interest. TaxPhlAn automatically designs primers for candidate markers, to generate SLST amplicons by PCR for subsequent marker gene sequencing. Finally, next generation sequencing (NGS) data containing SLST marker sequences can be processed in an automated workflow additionally offered by TaxPhlAn in module B of the pipeline (Suppl. Fig. S2). This oligotyping-based method analyses SLST amplicons sequenced by NGS, and, for each biological sample, calculates the relative abundances of SLST alleles corresponding to representative (sub)species of strains.

Although the era of metagenomics is fast approaching, with sequencing costs reducing by the day, the required data analysis depends on highly specialized pipelines and bioinformatics expertise. Furthermore, low biomass samples are not compatible for metagenomics sequencing. Therefore, the demand for more generic yet high resolution methods will remain. SLST analysis by TaxPhlAn will meet this demand by offering an additional (sub)species typing in parallel to genus level classification in one sample prepped for generic 16S sequencing. Hence, TaxPhlAn is no replacement of existing techniques such as 16S, but rather a complementary one.

TaxPhlAn is available as a Python/Perl command-line application, and is accessible through a pre-configured, plug-and-play Docker virtual machine which is supplied at the Docker Hub repository https://hub.docker.com/r/ederveen/taxphlan/. TaxPhlAn is supplied with test datasets, properly documented with an extensive user manual, and can conveniently be applied by anyone with limited bioinformatics knowledge.

The bacterial genus of *Staphylococcus* cannot reliably be profiled with high resolution using conventional 16S marker gene sequencing primers^13^, warranting the use of SLST. In this manuscript, we showcase a clinically relevant application of TaxPhlAn by profiling the presence of cutaneous bacteria (with 16S) as well as by targeted *Staphylococcus* species (with SLST) present in atopic dermatitis (AD) versus healthy skin of human volunteers. In short, our results show real-life and cost-effective application of the presented SLST methodology for profiling *Staphylococcus*, and demonstrate significant added value of SLST compared to 16S.

## Results

We have developed a bioinformatics pipeline that requires reference genomes as input, and then automates the discovery and design of gene targets and corresponding primer sets for SLST-based profiling of specific microbiota in complex communities (TaxPhlAn Module A: Suppl. Fig. S1). Primers designed by TaxPhlAn Module A can be applied to any biological sample to generate SLST marker gene sequences. We therefore additionally provide a data analysis workflow based on oligotyping to straightforwardly analyze SLST data (TaxPhlAn Module B: Suppl. Fig. S2). This analysis pipeline allows going from raw SLST marker gene sequencing data to a compositional microbiota-to-sample matrix, with corresponding heatmap visualization of the data and clustering of study samples.

### TaxPhlAn performs automated discovery of gene targets for application in SLST

We validated TaxPhlAn by evaluating four different bacterial taxa at the genus or species level that are typically found on two clinically relevant human niches: skin and gut. These are *Staphylococcus*, *Propionibacterium acnes*, *Bifidobacterium* and *Escherichia/Shigella*, representing both Gram-positive and Gram-negative genomes with variable sizes and GC-content (Table 1, and Suppl. Tables S1–S4 for more detailed information on each individual genome). For each of these four bacterial taxa we were able to successfully pinpoint multiple genic SLST targets able to distinguish (sub)species with significantly higher resolution than currently feasible with alternative high-throughput metataxonomics methods such as Illumina 16S marker gene sequencing on variable regions V1-V2 and V3-V4 (Table 2, and Suppl. Table S5 for primer information, and Suppl. Tables S6–S9 for TaxPhlAn reports).

**Table 1:** Datasets with bacterial genomes used for TaxPhlAn benchmarking.

Taxon Name	Taxonomy Level of Input	Gram-Staining	Human Niche	# Genomes selected	Average ± SD Genome Size (Mb)	Average ± SD Genome GC-content (%)
*Bifidobacterium*	genus	positive	gut	261	2.30 ± 0.27	60.1 ± 2.0
*Escherichia/Shigella*	supra-genus	negative	gut	200	4.91 ± 0.35	50.6 ± 1.1
*Propionibacterium acnes*	species	positive	skin	123	2.50 ± 0.03	60.1 ± 0.1
*Staphylococcus*	genus	positive	skin	200	2.59 ± 0.20	33.0 ± 1.4

For benchmarking we selected four bacterial taxa that are associated with the two clinically relevant human microbial niches of gut and skin. The datasets represent both Gram-positive and Gram-negative bacteria from different taxonomical levels, and with variable genome sizes and GC-content. See Suppl. Tables S1–S4 for more details. SD: standard deviation; Mb: mega base pairs.

**Table 2:** Performance of a default TaxPhlAn run versus common 16S regions based on the number of unique clusters identified.

Taxon Name	^#^SLST clusters			^#^16S V1-V2 clusters			^#^16S V3-V4 clusters		
	OUT Average ± SD	SDI Average ± SD	Coverage (%)	OTU	SDI	Coverage (%)	OTU	SDI	Coverage (%)
*Bifidobacterium*	42 ± 5	87 ± 8 (62)	95.8	10*	12 (56)*	5.0*	21	50 (26)	96.2%
*Escherichia/Shigella*	12 ± 6	29 ± 10 (11)	97.7	6	16 (8)	71.5	3	0 (0)	74.0%
*Propionibacterium acnes*	2 ± 1	7 ± 2 (16)	99.9	1	0 (0)	88.6	2	0 (0)	96.7%
*Staphylococcus*	24 ± 3	51 ± 6 (55)	91.1	12	41 (18)	94.0	5	9 (4)	100%

The number of reference genomes used on the benchmark can be found in Table 1, for an exact overview of these genomes see Suppl. Tables S1–S4. The number of unique SLST clusters found for each taxon-of-interest is based on the average of the top 10 SLST candidates from a default TaxPhlAn run (see also Suppl. Tables S6–S9). The number of 16S clusters found for these same taxa is based on primers 27 F and 338 R for V1-V2, and 357 F and 802RV2 for V3-V4 (V for 16S variable region) (Suppl. Table S5). Methods used for determining the number of clusters are either allele-based with a taxon-specific Shannon diversity index (SDI) threshold (in brackets the average number of informative SNP), or OTU-based with 97% identity threshold (accepted default in the microbiome field^48^). SDI thresholds were set to 0.6, except for *Propionibacterium acnes* for which we set the SDI to 0.2, because we observed less sequence variation on the level of species (for more information about SDI, we refer to the supplementary Methods). Values in brackets represent the average number of informative positions for an allele. Coverage is defined as the percentage of genomes identified with the primers tested. According to genome name annotations from our datasets, the number of species within the genus of *Bifidobacterium* is n = 46, *Escherichia/Shigella* n = 12, and *Staphylococcus* n = 29, and the number of strains within the species of *Propionibacterium acnes* is n = 123. SD: standard deviation, OTU: operational taxonomic unit, SDI: Shannon diversity index. *Note the very low coverage of V1-V2 primers for *Bifidobacterium*, in contrast to that of the V3-V4 primers.

### Orthologous, single-copy core genes allow for detection and typing of novel biodiversity

**Figure 2:**
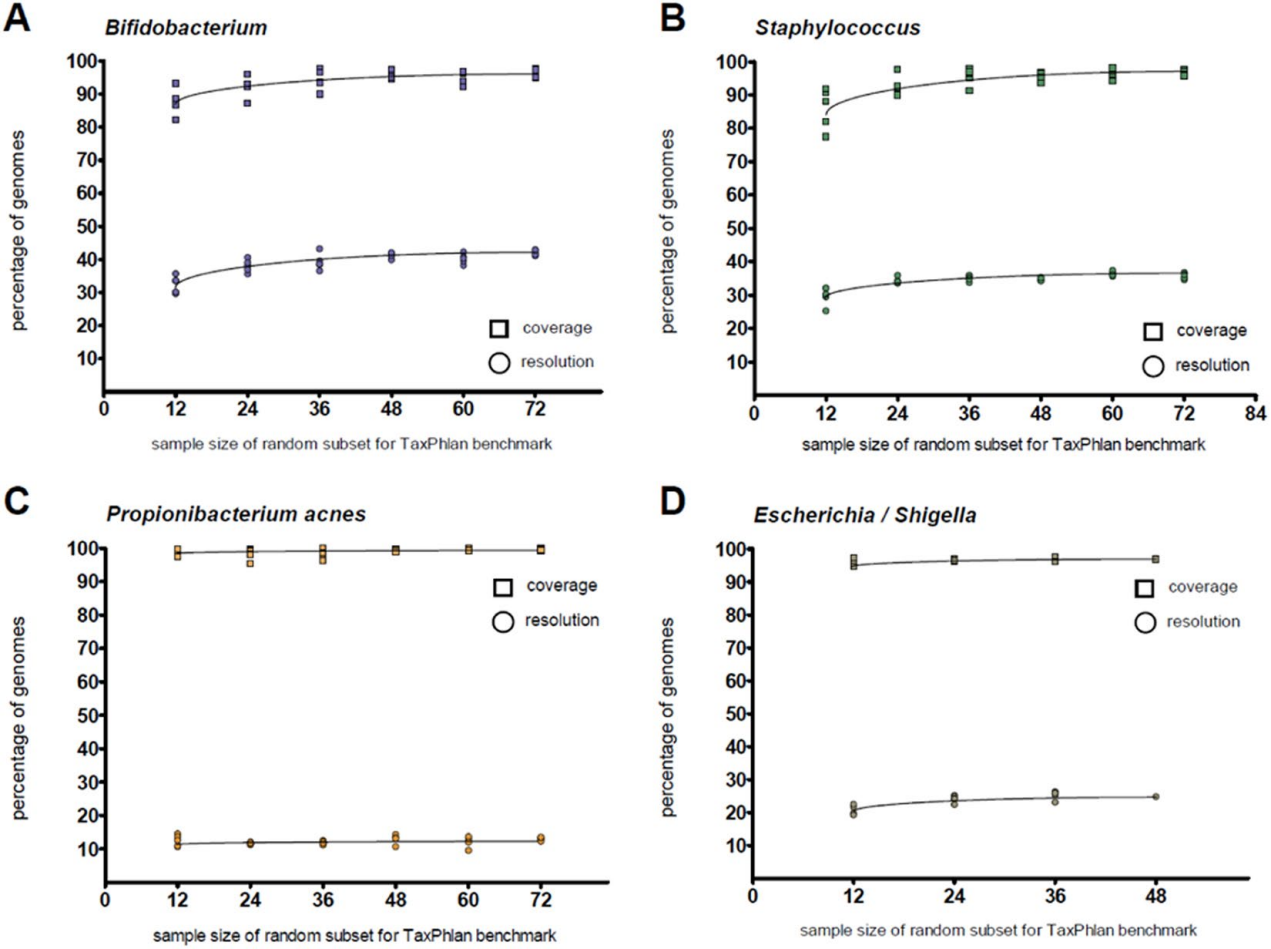
Reducing the number of input genomes for a TaxPhlAn run does not diminish performance of SLST candidates. We tested the stability, performance, and minimum number of input genomes required for the TaxPhlAn pipeline (Suppl. Fig. S1; Module A) by running the program with random subsets, and with variable sample sizes, of the total benchmark datasets. We determined for each run the number of unique SLST sequences (resolution, i.e. the number of phylotypes that can be distinguished using this amplicon in square symbols, expressed as percentage of total genomes for that dataset) and the total number of genomes for which an *in silico* PCR hit was predicted by PrimerProspector (coverage, in circle symbols). For each run, averages of the top 10 candidates were taken, and each run with each its unique random subset of genomes was plotted in the graph (i.e. 5 replicates). The maximum number of genomes in the benchmark datasets is 261 for *Bifidobacterium* (with datapoints in blue), 200 for *Staphylococcus* (green) and 200 *Escherichia/Shigella* (brown) and 123 for *Propionibacterium acnes* (orange).

TaxPhlAn relies on single-copy orthologous genes for discovery of SLST candidates (Suppl. Fig. 1, *Phase III*), in contrast to targets in intergenic regions, or orthologous genes that are not universally present. The advantage of this key SLST characteristic is that it makes it more likely that novel biodiversity is correctly identified, as we expect the SLST candidate genes to be universally present in the target taxon, and we expect the observed sequence variation in the SLST genes to correlate with the phylogeny (irrespective of its phylogenetic level). We tested this assumption *in silico* by running TaxPhlAn with random subsamples of the total genome datasets, for the four representative bacterial taxa as listed previously in Table 1. We undertook jackknifing by random subsampling of *n-*genomes from the total dataset with a step-size of 12 genomes, and running TaxPhlAn with default pipeline settings on that subset. Hereafter, the top 10 SLST candidates (i.e. primer sets) as reported by the pipeline were subjected to an *in silico* PCR on all available genomes in the entire dataset. The number of total genomes identified with an SLST target designed on a subset of 12 genomes did not improve significantly after addition of more genomes to this training set, nor did the number of unique SLST sequences identified (Fig. 2).

To confirm that high-scoring SLST targets as reported by the pipeline do indeed follow phylogeny based on full-genome information and hence contain an evolutionary conserved signal, we looked at the prime SLST candidates of each bacterial genome reference dataset. We observed that for every taxon-of-interest the variable region sequences of the prime SLST candidate correlated very well with the actual phylogenetic distances between genomes (based on SNP positions in the bacterial core genomes). This is illustrated by the unique SLST alleles capable of discriminating between different taxonomical clades, and as supported by their corresponding Spearman correlation values of 0.78 rho on average (for a projection of these alleles to the actual phylogenic tree, of each prime SLST candidate, see Suppl. Figs. S3–S6; for all predicted SLST targets for the reference runs and their phylogenetic distances correlation values see Suppl. Tables S6–S9). This data shows that TaxPhlAn allows for accurate phylogenetic placement of known taxa, and for inference of novel biodiversity, even when only a small number of genomes is available for design of an SLST target.

### TaxPhlAn significantly improves *Staphylococcus* profiling compared to 16S in clinical practice

**Figure 3:**
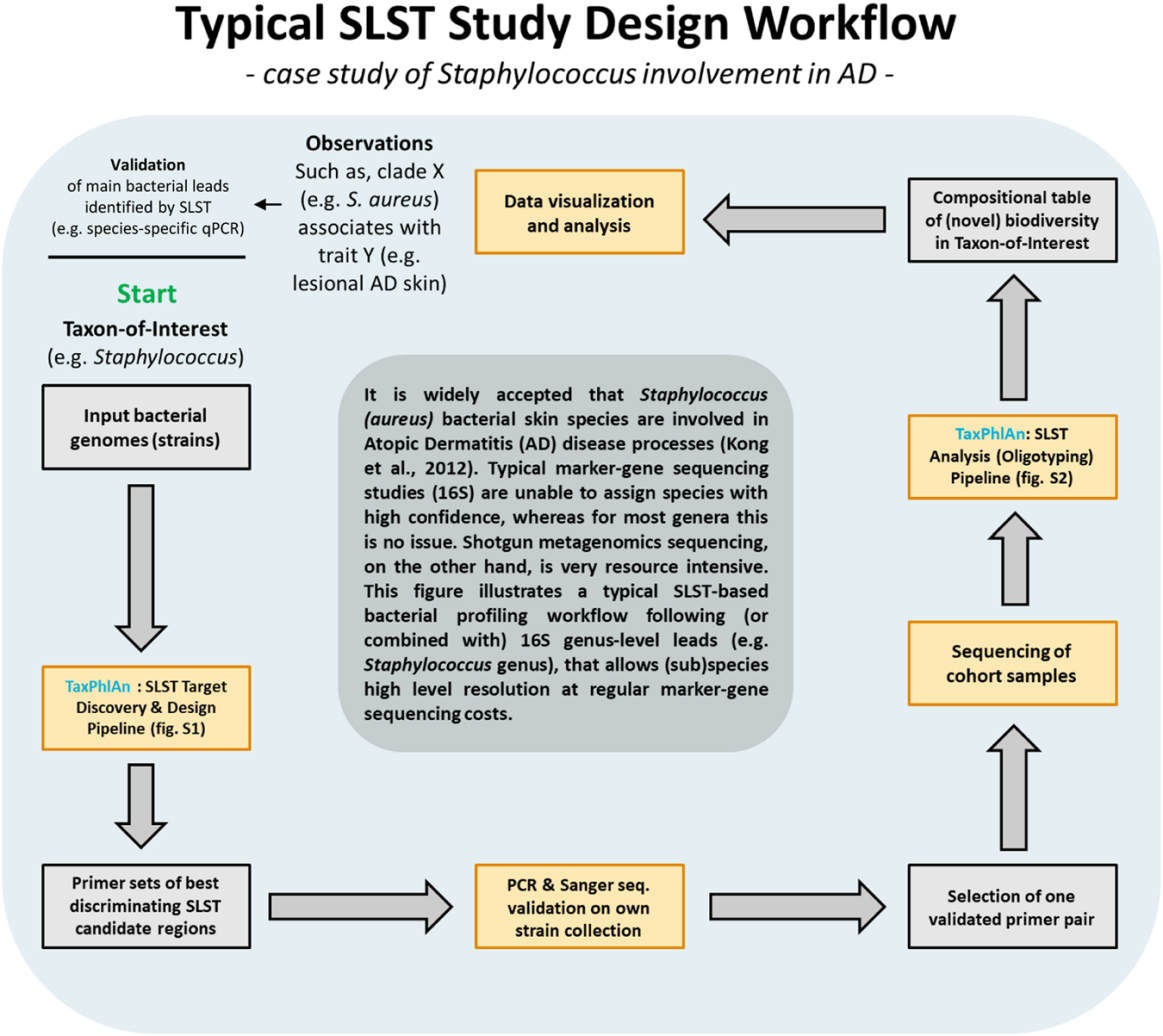
Workflow for SLST typing (graphical abstract of this AD substudy). This flow chart summarizes our proposed best practices for SLST. For this, we discuss the example of *Staphylococcus* involvement in AD as an SLST use case. It involves the selection of representative genomes for any taxonomy-of-interest, and analyzing them in a straightforward fashion with TaxPhlAn for Discovery & Design (Module A) of suitable SLST targets for optimal discrimination between input genomes. A small selection of the best-discriminating SLST candidates (i.e. primer pairs for PCR) will be tested in the laboratory by PCR on relevant strain collections. SLST candidates for which primer pairs survive both *in silico* and laboratory selection procedures can in principle be adopted as marker genes for typing of microbiota in next-generation sequencing (NGS) initiatives. Our TaxPhlAn SLST Oligotyping pipeline (Module B) allows for the analysis of SLST reads from NGS data, including typing of known and identification of unknown biodiversity, and reporting of results in comprehensible format, including automated visualization and clustering of samples. Altogether, this entire process facilitates the potential discovery of study parameters that associate with high-resolution microbiota data. Importantly, in order to “close the experimental circle”, microbiota leads from SLST data can be adopted in an attempt to unravel or further understand underlying biological processes. This is mainly based on pinpointing in high-resolution the most relevant microbial (sub)species, or even strains, for application in follow-up experiments or studies.

**Figure 4:**
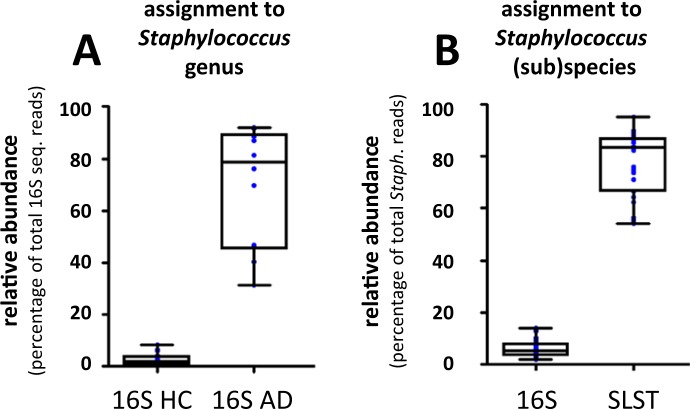
Significantly improved classification rate of *Staphylococcus* (sub)species for SLST compared to 16S. (**A**) Skin 16S V3-V4 sequencing data shows a significant increase for AD patients in *Staphylococcus* genus-level assigned reads in the dataset in comparison to HC (*p* = 0.002) (also see Suppl. Fig. S9), which is in line with literature^27^. (**B**) When evaluating the percentage of (sub)species-level assigned reads, that is, number of total *Staphylococcus* reads that could be classified to species level or lower, we observe a significant increased classification rate for SLST in comparison to 16S (*p* < 0.0001). Boxplots as median with interquartile range and whiskers from min to max. Blue dots represent individual data points.

The Gram-positive bacterium *Staphylococcus* represents a historically relevant genus in relation to AD. *Staphylococcus aureus* associates strongly with AD disease onset and severity^25–28^, whereas *S*. *epidermidis*, amongst others, is considered a skin commensal^29^. However, in general, the 16S rRNA gene does not allow for confident classification of bacteria to the level of species^12^. TaxPhlAn was designed to increase the resolution of bacterial profiling in clinical practice. We therefore compared SLST with 16S rRNA gene sequencing for classification of *Staphylococcus* species. In order to corroborate application and feasibility of the TaxPhlAn SLST method and corresponding proposed experimental workflow (Fig. 3), and to demonstrate clinical relevance, we enrolled from our hospital dermatology clinic a group of patients diagnosed with AD (n = 5), and healthy controls (HC) without a history of skin diseases (n = 9) (Suppl. Table S10). The skin microbiota on the left and right antecubital fossa (inner elbow) of these human volunteers was obtained by a standardized wet swabbing method, from normal and lesional skin for HC volunteers and AD patients, respectively. Skin bacteria were typed by 16S metataxonomics (V3-V4), and *Staphylococcus* species were determined by SLST marker gene sequencing (Suppl. Table S5 for 16S and SLST primer information). To confirm that we have a representative AD study cohort, we first analyzed the skin microbiota on the level of genus by 16S (Suppl. Table S11 for 16S sequencing read statistics), and found that *Staphylococcus* dominates skin of AD patients at the expense of *Propionibacterium* (Fig. 4A, and Suppl. Fig. S9, which is in line with literature^27^).

Based on *Staphylococcus* full-length 16S sequences (V1-V9) and clustering of these sequences into OTUs with 97% identity (Suppl. Table S12), no discrimination can be made between clinically relevant species. This analysis of V1-V9 identified only two major clusters, and these do not correlate well with phylogeny, as indicated by their ambiguous distribution within *Staphylococcus* subclades such as *S*. *aureus* and *S*. *haemolyticus* (Suppl. Table S12). For 16S sub-regions V1-V2 and V3-V4, species of *S*. *epidermidis* and *S*. *capitis* cannot be distinguished, albeit that these are in fact very different species (Suppl. Table S12). Likewise, *S*. *aureus* cannot be distinguished from *S*. *schweitzeri* and *S*. *argenteus* species for V1-V2 and V3-V4. Furthermore, *S*. *aureus* and *S*. *epidermidis* species can only be discriminated by V1-V2, and not by V3-V4.

By adopting the TaxPhlAn Discovery & Design pipeline on the *Staphylococcus* reference dataset as listed in Table 1 (Suppl. Fig. S1; Module A), we identified an SLST target that allowed for best discrimination of *Staphylococcus* species, including multiple sub-species clades. This SLST target, denoted as orthologous group (OG) #1123 (Suppl. Fig. S7), shows a higher resolving power than 16S regions V1-V2 and V3-V4 (Table 2). OG #1123 was selected as our prime SLST candidate after elaborate *in silico* (Suppl. Table S13) and laboratory validations (Suppl. Fig. S8) to prove specificity, and to exclude cross-reactivity with phylogenetically distant bacteria. OG #1123 is predicted to be part of the 30 S ribosomal protein S11 (note, this is not an rRNA gene), with an on average amplicon sequence length of 381 nucleotides (including primers). We performed marker gene sequencing of OG #1123 on the AD study cohort, and analysis of the obtained sequence data by the TaxPhlAn SLST Oligotyping pipeline (Suppl. Fig. S2; Module B). We found that the SLST target allowed for 77.2% of *Staphylococcus* reads to be assigned to species-level or lower. In comparison to 5.9% for 16S (Fig. 4B), which is a tremendous improvement in *Staphylococcus* classification for SLST over conventional 16S-based sequencing approaches.

### TaxPhlAn allows for profiling of *Staphylococcus* phylotypes in complex patient communities

**Figure 5:**
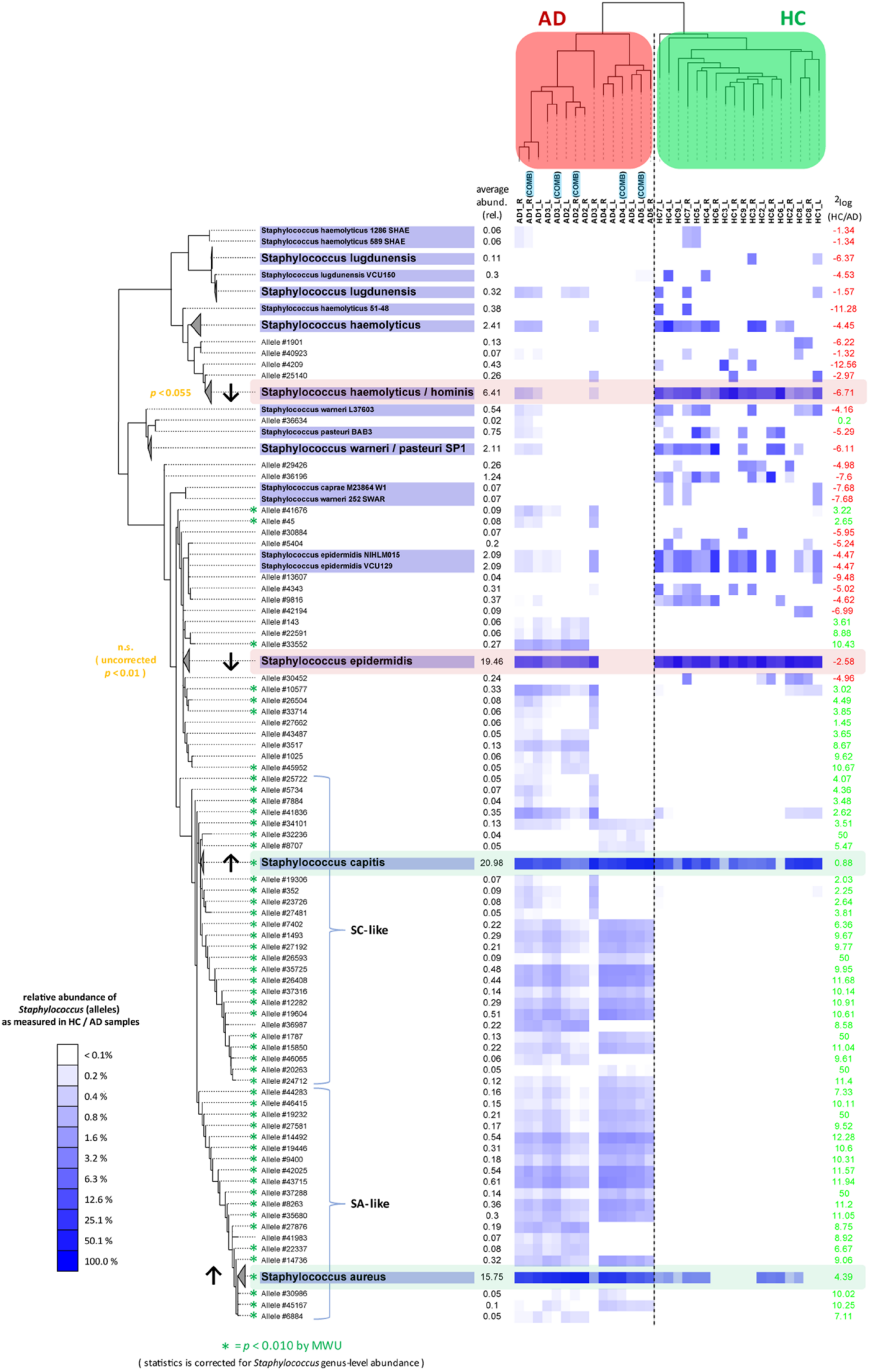
*Staphylococcus* SLST data reveals two distinct clusters separating HC and AD samples, typed by *S*. *aureus*- and *S*. *capitis*-like species. Heatmap summarizing the SLST *Staphylococcus* data from AD patients and HC volunteers (Suppl. Table S14). The SLST data was analyzed by the TaxPhlAn Oligotyping pipeline (Module B). The tree on the left is the maximum-likelihood phylogenetic tree of the SLST allele sequences generated with FastTree^47^. The alleles were built with an Shannon diversity index (SDI) threshold of 0.6, resulting in 45 SNP positions of the SLST marker gene sequences. Alleles for which a reference genome was available were named after this reference (e.g. *S*. *epidermidis* VCU129), instead of having an allele number (e.g. *Allele #30452*). Multiple references were collapsed into one clade if they shared the same allele sequence (e.g. *S*. *epidermidis*). The heatmap represents log10-transformed SLST relative abundances. A green asterisk indicates significantly differentially abundances, according to a MWU test, and corrected for *Staphylococcus* genus-level relative abundance derived from 16S data (Suppl. Table S15). Note that the phylogenetic tree was cropped at top and bottom in order to facilitate visualization, leaving out some lowly abundant and statistically insignificant taxa from this view (for a full list see Suppl. Table S14). Sample labels in blue are samples where 16S and SLST marker gene PCR amplicons of the same sample were pooled (technical replicates). SA- and SC-like: *S*. *aureus*- and *S*. *capitis*-like.

In further analysis of the *Staphylococcus* SLST data of the AD cohort, we observed a strong and significant increase of allele clusters classified as *S*. *aureus* in AD patients relative to healthy controls (30.07% AD, 1.43% HC, *p* = 0.006), and a significant decrease of *S*. *epidermidis* (5.58% AD, 33.34% HC, *p* = 0.006) and *S*. *haemolyticus/S*. *hominis* (0.12% AD, 12.70% HC, *p* = 0.002) allele clusters in these patients (Fig. 5, and Suppl. Table S14 for the corresponding SLST data). Although there is a large difference in the average level of *S*. *capitis* between the two study groups, this difference is not statistically significant (27.22% AD, 14.74% HC, *p* = 0.25).

Next, one can also study the SLST data in context of the known *Staphylococcus* genus-level relative abundances based on 16S, because both SLST and 16S were measured for each sample. This was done for each SLST allele by multiplication of its relative abundance value to that of *Staphylococcus* 16S of the same sample. When taking into account these *Staphylococcus* relative abundances according to 16S then the aforementioned shift of SLST *S*. *epidermidis* and *S*. *haemolyticus/S*. *hominis* clusters between the experimental groups is lost, but not for *S*. *aureus* (Suppl. Fig. S10, and Suppl. Table S15 for the corresponding SLST data including/corrected for 16S). This is possibly a result of the low relative abundances of *Staphylococcus* in HC samples. Most interestingly, a statistically significant increase of *S*. *capitis* in AD patients is observed when taking *Staphylococcus* 16S genus-level relative abundances into account (19.40% AD, 0.47% HC, *p* = 0.003) Suppl. Fig. S10B).

Redundancy analyses (RDA) of the AD cohort further substantiates that skin microbiota profiles from HC and AD individuals are significantly different, both for 16S- and SLST-based sequencing data (Suppl. Figs. S11–S12; *p* = 0.02). Although RDA on 16S species- and OTU-classified data both indicate that *S*. *aureus* is crucial for separation of HC and AD subjects (Suppl. Fig. S11A,B, respectively), we observe that 16S simply does not recognize most of the OTUs as *S*. *aureus* (likewise for *S*. *epidermidis*). We hereafter combined 16S with SLST data in one analysis, i.e. SLST allele abundances were set relative to the total bacterial population, not relative to only the *Staphylococcus* fraction. Interestingly, this preserves the significant signals that allow for separating HC from AD, and also substantially increases the classification rate of *Staphylococcus* species, thereby showcasing the added value of such approach (Suppl. Fig. S12B). Most notably, the combined RDA shows a great importance for *S*. *aureus* (22.75% in AD) and *S*. *capitis* (19.40%) in discriminating between the two study groups, which is in line with the univariate analyses as reported earlier in Suppl. Fig. S10A,B.

The recurring associations of *S*. *capitis* with AD disease prompted us to experimentally validate these findings by an alternative approach. For this, we performed qPCR on *S*. *capitis* with species-specific primers^30^ and measured their levels in our cohort of HC and AD samples (Suppl. Figs. S13 and S14). Once again, we observe significantly higher levels for *S*. *capitis* in AD relative to healthy controls (*p* = 0.004) together with qPCR levels that correlated very strongly with SLST levels (Spearman rho = 0.92, *p* < 0.0001), thereby replicating the exact same biological effect as observed by SLST (Suppl. Fig. S15).

Finally, 16S and SLST sequencing efforts were performed separately, however, five 16S technical replicates were taken along in the SLST sequencing run. These technical replicates show highly similar profiles, even though their 16S and SLST amplicons were pooled into one sequencing sample (Fig. 5, and Suppl. Fig. S9; samples indicated in blue are the pooled technical replicates). Likewise, biological replicates cluster very well, indicating that there is little intra-individual difference between sampling arms of the same volunteer.

## Discussion

SLST marker gene sequencing has recently been described for determining strain level identification of specific bacteria within a microbiome^21^. Other methods for bacterial typing or profiling, such as species-specific PCR approaches or DNA fingerprinting-based methods for identification, usually do not have the desired resolution to pinpoint strains of interest, are not compatible with high-throughput screening and/or are very labor intensive. TaxPhlAn fills the gap between the relatively cheap but coarse-grained 16S rRNA-based analyses and the more expensive, complex and laborious but higher resolution metagenomics sequencing.

TaxPhlAn SLST profiling can be efficiently combined with a 16S amplicon approach, either following-up on an initial 16S pilot study that generated microbial leads in order to zoom-in on that clade with a higher resolution than feasible by 16S, or directly, by combining 16S and SLST amplicons in one single marker gene sequencing run. Our experimental data show that combining 16S and SLST amplicons for sequencing is practically feasible, can be done without adverse loss of sequencing depth, and without affecting microbiota profiles as obtained by separately sequencing 16S and SLST amplicons. This is particularly relevant, as it enables running SLST experiments in tandem with regular 16S sequencing efforts without additional sequencing costs. SLST sequencing can be multiplexed, for example by combining multiple different SLST targets per sample in a single sequencing initiative, thereby boosting either the discriminatory resolution of SLST (hence, in principle approaching MLST marker gene sequencing), or enabling the profiling of multiple bacterial taxa in the same experiment.

As an additional feature, upon running the TaxPhlAn SLST Discovery & Design pipeline one is able to assign genomes-of-interest (strains) in order to filter on SLST candidates that allow distinguishing these genomes from all other input genomes. This is particularly useful when there is specific need to identify one particular phylotype in your samples, for example when one needs to distinguish sub-species *Bifidobacterium longum subsp*. *longum* from *Bifidobacterium longum subsp*. *infantis*. Likewise, one is able to assign negative control genomes to filter on primers of SLST candidates that do not recognize these genomes, for example when one needs to have SLST candidates of which the primers are specific for *Propionibacterium acnes*, but not for other closely related commensals such as *Propionibacterium namnetense*.

One limitation of DNA amplification-based approaches for profiling of specific taxonomic groups is the requirement of sequenced (reference) genomes in order to identify suitable molecular targets. 16S marker gene sequencing does not necessarily require reference genomes to identify new biodiversity, because it is based on the 16S rRNA gene that is universally present and conserved in all prokaryotes, and these sequences correlate well with phylogeny. For correct taxonomic classification of 16S marker gene sequencing reads prior knowledge from 16S rRNA gene databases is required, e.g. RDP^31^, GreenGenes^32^ and SILVA^33^. For SLST these considerations are similar to those of 16S. Even though TaxPhlAn requires a set of references, a dozen of sequenced reference genomes is sufficient to allow for confident inference of phylogenetic information as shown by the benchmarking results presented in this study. Nonetheless, the more reference genomes available for oligotyping analysis, the more confident SLST allele classification and inference of novel biodiversity will be. Yet, although our demonstrated maximum achievable resolution for SLST is an improvement over 16S (Fig. 2, Table 2), one has to keep in mind that this resolution is also dependent on the phylogenetic complexity of the specific bacterial taxon under study (but that of course also applies to 16S marker genes). Also, although we here presented a generic methodological workflow applied to bacteria only, we see no theoretical objections for the characterization of archaea, fungi or lower unicellular eukaryotes with a similar SLST approach.

In the current study, we mainly identified taxa of *S*. *aureus* and (to a lesser extent) *S*. *capitis*, which were significantly increased in AD, in comparison to taxa of *S*. *epidermidis* and a cluster of *S*. *haemolyticus/S*. *hominis*, which were associated with healthy skin. Although these are known *Staphylococcus* species, also many *S*. *aureus*-like and *S*. *capitis*-like unknown taxa were identified which were significantly more abundant in AD, for which no existing reference is available in current databases, but for which we now know their phylogenetic relation to known *Staphylococci*. Importantly, the relation of *S*. *capitis* with AD disease as found by our SLST method has to our knowledge not been reported before, except for one study by Byrd *et al*. that reports the presence of *S*. *capitis* in AD lesions together with *S*. *aureus*, *S*. *epidermidis* and *S*. *hominis* (using a multiplex PCR assay)^34^. This therefore compels us to take our findings on *S*. *capitis* in relation to AD with caution given our limited sample size and lack of more extensive validations. Furthermore, the correlation of *S*. *capitis* with AD is statistically significant only when SLST relative abundances have been corrected for the corresponding *Staphylococcus* (genus-level) abundance for each sample based on 16S (Suppl. Tables S14 and S15), whereas this is not the case for *S*. *aureus*. Nevertheless, we were able to replicate these findings by an alternative method with targeted qPCR using *S*. *capitis*-specific species primers. Although the calculated relative levels of *S*. *capitis* by qPCR differ in extent to those by SLST we reported a very strong correlation between these values. We believe it is to be expected that these values will not be one-to-one comparable because of inherently different methods used, (primer) efficiencies, 16S copy number variation, etc. Although it may seem a less likely scenario, it should also be mentioned that former studies might have missed this *S*. *capitis* species because of the limited resolution of 16S, or because of the fact that previous in-depth metagenomics studies on AD did not include lesional skin samples^28^. Of final relevance to this discussion about *S*. *capitis* and its relation to AD, is a study by Cameron *et al*., 2015 that revealed an arsenal of virulence factors in the genome of *S*. *capitis* that likely contribute to its pathogenicity^35^, which makes it appealing to hypothesize that this species could potentially have a role in AD. Nonetheless, although we show in this study that SLST provides insight into specific microbiota at higher taxonomical detail than 16S, and allows novel microbial candidates to be detected: these microbial leads (similar to 16S) still require ultimate validation by alternative methods, such as by targeted PCR, before designing follow-up experiments for mechanistic understanding of a microbe’s role in any system.

In conclusion, TaxPhlAn provides a method for the automated design of SLST amplicons and the analysis of SLST sequencing data. As TaxPhlAn evaluates regions that are single-copy orthologous genes, the resulting SLST amplicons allow detection of novel variants and placement of these variants in phylogenetic context.

## Methods

### Skin microbiome sample collection and processing

In advance of study start, collection of human material (skin samples by non-invasive skin swabbing procedures) was approved by the local medical ethical committee named: Radboudumc Commissie Mensgebonden Onderzoek (CMO) Arnhem-Nijmegen, and individual written informed consent from the volunteers were obtained. The study was performed according to the Declaration of Helsinki principles. Microbiome skin samples were collected by a wet swabbing protocol of the inner elbow (antecubital fossa) skin of human healthy control (HC) volunteers (n = 9) and atopic dermatitis (AD) patients (n = 5). Inclusion criteria (and exclusion, with exception of having AD) as described by Zeeuwen *et al*.^36^. All human volunteers were enrolled in the dermatology clinic of the Radboud University Medical Center, Nijmegen, and AD was diagnosed by a trained dermatologist. Sample collection was performed as described previously^14,36^. In short, a 4 cm^2^ skin area of the inner elbow was swabbed with sterile Catch-All sample collection swabs (Epicentre Biotechnologies, Madison, USA). The swabs were soaked in sterile SCF-1 solution (50 mM Tris buffer [pH8], 1 mM EDTA, and 0.5% Tween-20) before sample collection. Mock swabs, only exposed to ambient air, were taken as negative controls. The Mobio Ultraclean Microbial DNA isolation kit (Mobio laboratories, Carlsbad, USA) was used according to the manufacturers protocol to extract microbial DNA, before storing it at −80 °C until further use.

### 16S and SLST PCR chemistry and conditions

Primer sequences used for sequencing of the 16S V3-V4 region or by SLST can be found in Suppl. Table S5, and were appended with Illumina adaptor sequences and sample-specific barcodes. PCR protocol with KOD hot start DNA polymerase as follows: 2 m 95 °C hot start; 35 cycles of 20 s 95 °C, 10 s 61 °C, 15 s 70 °C; 10 m 70 °C. PCR quality control as follows: agarose gel amplicon sizes were checked, and Sanger sequenced to validate with BLAST to the NCBI database. SLST primer specificity was tested on the following *Staphylococcus* strains: *S*. *aureus* (ATCC 29213), S. *epidermidis* (ATCC 12228), and clinical isolates of *S*. *capitis* and *S*. *hominis*. As negative controls we used the common skin commensals: *Propionibacterium acnes* (ATCC 6919), *Pseudomonas aeruginosa* (ATCC 27853), *F*. *magna* (ATCC 15794), and a clinical isolate of *C*. *aurimucosum*.

### 16S and SLST marker gene sequencing and data pre-processing

PCR 16S and SLST amplicon libraries were generated as described above. For each of the 5 AD patients, one additional pooled sample was taken along, with mixed 16S and SLST amplicons. The libraries were barcoded, multiplexed and sequenced on an Illumina MiSeq machine with paired-end 300 cycles protocol and indexing, by BaseClear B.V. (Leiden, The Netherlands). 16S and SLST sequencing data were generated in separate Illumina runs, but the 16S/SLST pooled samples (technical replicates) were taken along with the SLST run. Illumina sequencing data was quality checked and demultiplexed by BaseClear standards, as detailed in the supplementary Methods, and FASTQ files were generated. Paired-end reads were assembled into pseudoreads with PEAR^37^, with strict assembly settings: quality threshold 30, minimum overlap 35 and *p-*value 0.0001. On average, only 0.17% of the raw reads could not be assembled (data not shown). Thereafter, for the 16S/SLST pooled samples, the pseudoreads were split to 16S or SLST input FASTA files by a local BLAST to a SLST gene database of the 7078 available *Staphylococcus* genomes: query reads with a hit (by default BLASTn settings from version 2.2.29 + ) were used for SLST analysis, if not send for 16S analysis by QIIME. This script has been made available as ‘*split-reads-to-16S-SLST’* in the TaxPhlAn Docker image.

### 16S marker gene sequencing data analysis

For generation of the 16S-derived taxa-to-sample compositional matrix, a customized Python workflow based on Quantitative Insights Into Microbial Ecology (QIIME version 1.8)^38^ was adopted (http://qiime.org). Reads were filtered for chimeric sequences using the UCHIME algorithm version 4^39^. Hierarchical clustering of samples was performed using UPGMA with weighted UniFrac as a distance measure as implemented in QIIME 1.8. Figures resulting from these clustering analyses were generated using the interactive tree of life (iTOL) tool^40^. The Ribosomal Database Project classifier version 2.3 was performed for taxonomic classification of the sequence reads^41^. Alpha diversity metrics (PD whole tree, Chao1, Observed Species and Shannon) were calculated by bootstrapping 6490 reads per sample, and taking the average over 10 trials. For visualization of the differential microbiome, Cytoscape software version 3.4.0^42^ was used together with in-house developed Python scripts for generating the appropriate input data deriving from the QIIME analysis. Note that due to technical limitations in the resolution of 16S marker gene sequencing, OTU (operational taxonomic unit) calling on the level of species should be interpreted with caution.

### Selection of representative *Staphylococcus* genomes for TaxPhlAn target discovery

*Staphylococcus* genomes were downloaded (n = 7247) from the NCBI assembled genomes database^43^ (ftp.ncbi.nlm.nih.gov/genomes/genbank/bacteria/) and the NCBI Traces WGS database (https://www.ncbi.nlm.nih.gov/Traces/wgs/). TaxPhlAn requires sub-selection of input genomes due to computational capacity. We selected a diverse subset of those genomes based on their 16S sequences by running a BLAST search with the full 16S gene of *S*. *aureus subsp*. *aureus* 71193 to each available genome (*Staphylococcus* 16S reference gene was downloaded from SILVA database^33^ in April 2016; accession CP003045). Based on the best scoring BLAST hits, 16S genes were aligned to the aforementioned 16S reference gene by a pairwise global alignment with Needle (version EMBOSS: 6.3.1; default settings)^44^. From these alignments, a 1Kb long 16S region covered by the majority of genomes was selected (n = 6569) and 16S OTU clusters were determined using UCLUST (version 1.2.22q)^39^ for cluster building with percentage identity set to 99.7%. This yielded 24 (16S) clusters from which we equally selected 200 unique *Staphylococcus* genomes in total. For further details we refer to the supplementary Methods.

### TaxPhlAn (Module A): SLST target discovery and design workflow

TaxPhlAn is an acronym for SLST-based **Tax**onomy **Ph**y**l**ogenetic **An**alysis, and it is made up of two connected pipelines (Modules A and B). The TaxPhlAn Discovery & Design pipeline (Module A) finds SLST markers of single locus orthologous genes to profile specific bacterial taxa up-to and beyond the species level, based on a set of reference genomes provided by the user. It consists of a series of Perl/Python scripts, and has been made available through the supplied Docker as ‘*TaxPhlAn-SLST-Design-wrapper*.*py’* (wrapper script). For more details concerning the TaxPhlAn Discovery & Design workflow, we refer to the supplementary Methods. In short, it consists of the following steps (Suppl. Fig. S1). *Initiation and QC:* (*Phase I-a*) data preparation based on a configuration file and input genomes; (*Phase I-b*) data preprocessing by input file reformatting and genome annotation. *Orthology:* (*Phase II*) orthology calculations and phylogenetic analysis on provided genomes. *Target Discovery and Design:* (*Phase III-a*) selection of core gene clusters for candidate marker genes; (*Phase III-b/c*) prediction of variable regions in candidate core clusters. *Validation and Reporting:* (*Phase IV*) *in silico* primer design and evaluation of candidate variable regions, and final reporting of results.

### TaxPhlAn (Module B): SLST data analysis (oligotyping) workflow

The TaxPhlAn Oligotyping analysis pipeline (Module B) is used to analyze (and visualize) raw SLST marker gene sequences from raw sequencing data to a compositional microbiota-to-sample matrix (Suppl. Table S14). It consists of a series of Python scripts, and has been made available through the supplied Docker as ‘*TaxPhlAn-SLST-Oligotyping-wrapper*.*py’* (wrapper script). For more extensive details concerning the TaxPhlAn *oligotyping analysis* workflow we refer to the supplementary Methods. In short, it consists of the following summarized steps (see graphical outline in Suppl. Fig. S2). *Initiation and QC: (Phase 1)* Data preparation with mandatory input and quality control. *Allele building: (Phase II)* Oligotyping by allele building based on Shannon diversity index. *Allele matching: (Phase III-a)* SLST allele matching and scoring*; (Phase III-b)* Phylogenetic analysis of alleles, and data visualization. *Reporting: (Phase IV)* Oligotyping data results and reporting.

### Statistics

For the microbiota data in this manuscript, statistical significance between contrasts with regard to taxonomy abundances was tested by a non-parametric (unpaired) Mann-Whitney U (MWU), uncorrected. Statistical tests were performed by custom, in-house Python scripts (SciPy module version 0.17.0; https://www.scipy.org/) downstream of QIIME. Redundancy Analysis (RDA) was done using Canoco 5.04^45^ using default settings of the analysis type “Constrained”. Relative abundance values for taxa were used as response data, and the sample classes as explanatory variables. RDA calculates *p-*values by permuting the sample classes. Correlations were examined using Spearman’s rank test as performed by custom, in-house R scripts (version 3.2.2; https://www.r-project.org/). For any other type of data visualization we adopted GraphPad Prism 5.0 or Microsoft Office Excel 2016. Significances mentioned in figures are as follows: n.s. (not significant), *p < 0.05, **p < 0.01, ***p < 0.001.

### Sequencing data availability

SLST and 16S Illumina sequencing data is available for download at the European Nucleotide Archive (ENA) database (http://www.ebi.ac.uk/ena)^46^ under study accession number PRJEB27442 (or secondary accession number ERP109520). The sequencing data is available in FASTQ-format, including corresponding metadata for each sample. An overview of the samples and metadata can be found in Suppl. Table S10.

### TaxPhlAn pipeline distribution

TaxPhlAn is accessible through a pre-configured, plug-and-play Docker virtual machine which is supplied at the Docker Hub repository: https://hub.docker.com/r/ederveen/taxphlan/. Directions to SLST test datasets and documentation can be found in the TaxPhlAn Docker home directory upon running the Docker image. For quick and easy install of the TaxPhlAn Docker image please see GitHub at: https://github.com/ederveen/taxphlan/.

## Supplementary information

Supplementary Information

Supplementary Tables S1-S4

Supplementary Tables S6-S9

Supplementary Table S11

Supplementary Table S12

Supplementary Tables S14-S15

## Data Availability

SLST and 16S Illumina sequencing data is available for download at the European Nucleotide Archive (ENA) database (http://www.ebi.ac.uk/ena) under study accession number PRJEB27442. TaxPhlAn is accessible through a pre-configured, plug-and-play Docker virtual machine which is supplied at the Docker Hub repository: https://hub.docker.com/r/ederveen/taxphlan/.
