# Supplementary material for: A generic workflow for Single Locus Sequence Typing (SLST) design and subspecies characterization of microbiota
Source: Sci Rep. 2019 Dec 27;9:19834. doi: 10.1038/s41598-019-56065-y (PMC6934516; doi:10.1038/s41598-019-56065-y)

Supplementary Methods

A generic workflow for Single Locus Sequence Typing (SLST) design and subspecies characterization of microbiota.

Thomas H.A. Ederveen 1-3 #, Jos P.H. Smits 2, Karima Hajo 1, Saskia van Schalkwijk 3, Tessa A. Kouwenhoven 2, Sabina Lukovac 3, Michiel Wels 1,3, Ellen H. van den Bogaard 2, Joost Schalkwijk 2, Jos Boekhorst 1,3, Patrick L.J.M. Zeeuwen 2 and Sacha A.F.T. van Hijum 1,3

1. Center for Molecular and Biomolecular Informatics (CMBI), Radboud Institute for Molecular Life Sciences (RIMLS), Radboud University Medical Center (Radboudumc), Nijmegen, The Netherlands.

2. Department of Dermatology, RIMLS, Radboudumc, Nijmegen, The Netherlands.

3. NIZO, Ede, The Netherlands.

# Corresponding author: Thomas H.A. Ederveen, Center for Molecular and Biomolecular Informatics (CMBI), Radboud Institute for Molecular Life Sciences (RIMLS), Radboud University Medical Center (Radboudumc), Geert Grooteplein Zuid 28 (route 260), 6525 GA, Nijmegen, The Netherlands. E: Tom.Ederveen@radboudumc.nl, T: 0031-243619390

1. Selection of representative *Staphylococcus* genomes for TaxPhlAn target discovery

All genomes classified as *Staphylococcus* (n = 7247; 2016 Apr.) were downloaded from the NCBI assembled genomes database [1] using a custom in-house python script (`ftp.ncbi.nlm.nih.gov/genomes/genbank/bacteria/`) and from the NCBI Traces WGS database (`https://www.ncbi.nlm.nih.gov/Traces/wgs/`). The genomes with status (RefSeq category annotation) '*reference*' or '*representative*' and those with a genome length of 2-3Mb (2.8Mb on average) and with no more than 300 contigs / scaffolds were selected for analysis (n = 7085). The TaxPhlAn pipeline requires a sub-selection of input genomes due to computational capacity (n = 200-250 max). Therefore, we selected those genomes representing the largest degree of genomic differences in the *Staphylococcus* genus based on their 16S sequences as follows: from the filtered *Staphylococcus* genomes, 16S genes were determined by BLAST [2] with as query the full 16S gene of *S. aureus subsp. aureus 71193*. This sequence was downloaded from SILVA database [3] in April 2016; accession CP003045; this sequence was selected based on the longest available gene length (1831nt), in combination with the highest available sequence-, alignment- and pintail quality, which are 95.61%, 99.91% and 100.00%, respectively. As 16S genes are typically present in multi-copy in most bacterial genomes, for each genome only the hit with lowest E-value was evaluated. All genomes with a valid BLAST hit were considered (n = 7073), and for genomes with a minimum BLAST hit length of 1Kb the corresponding 16S region was used for further phylogenetic analysis (n = 6985). The selected 16S genes were aligned to the aforementioned 16S reference gene by a pairwise global alignment with Needle (version EMBOSS: 6.3.1; default settings) [4]. Based on these alignments, the 16S alignment region with the highest number of genomes covering at least 1Kb sequence was selected (n = 6569): this is position 296 to 1303 of the reference. 16S OTU clusters were determined using UCLUST (version 1.2.22q) [5] with percentage identity set to 99.7% (stepwords value set to 1007, in line with selected region length) which yielded 24 different clusters. From these 24 clusters we selected 200 unique *Staphylococcus* genomes in an automated manner, based on: (**i**) species annotation (i.e. taking along at least one genome per unique species for each cluster), (**ii**) genome status (i.e. reference /

representative, closed / complete, or draft genome with ascending *n*-contigs or *n*-scaffolds, prioritizing in that order) as provided by the NCBI genome database [1], and (**iii**) with a minimum of one genome per cluster and a maximum of 10 genomes, for a total of 200 genomes.

2. Illumina sequencing data quality checking and demultiplexing

16S and SLST marker gene sequencing and collection of raw sequencing data was performed as detailed in the main manuscript. The sequencing run was analyzed with the Illumina CASAVA pipeline (v1.8.3) with demultiplexing based on sample-specific barcodes. The raw sequencing data produced was processed removing the sequence reads of too low quality (only 'passing filter' reads were selected) and discarding reads containing adaptor sequences or failing PhiX Control with an in-house filtering protocol. A quality assessment on the remaining reads was performed using the FASTQC quality control tool version 0.10.0 [6].

3. TaxPhlAn (Module A): SLST target discovery and design workflow (see Suppl. Fig. S1)

3.1 Data preparation phase: Configuration file and input genomes. (Phase I-a)

In order to use the TaxPhlAn SLST design pipeline (‘*TaxPhlAn-SLST-Design-wrapper.py*’), users are asked to provide a configuration file (i.e. *config.txt*) which should consist of the user-provided (private or public) genomes: detailing (**i**) their file name, (**ii**) genome identity (preferably strain-level annotation, if available), and (**iii**) status of the genome. Genome status can be ‘*ingroup*’ for known ingroup genomes-of-interest, ‘*outgroup*’ for a selection of known outgroup strains (representatives, preferably) and are used to identify SLST amplicon primers only recognizing the taxa in the ingroup, or ‘*extra*’ for any remaining available genome of closely related stains and / or with unknown phylogenetic placement. We furthermore provide the users the ability to assign ‘*strain-of-interest*’ for one or multiple genomes that one wishes to explicitly distinguish from other input and ‘*strain-of-interest*’ genomes. Note that a ‘*strain-of-interest’* is automatically treated as an ingroup genome, and the pipeline will provide information on candidates that best discriminate that genome from other genomes-of-interest. Please be advised that due to computational intensity it is not recommended for the total number of input genomes to exceed a maximum of 200-250 ‘*ingroup*’ or ‘*strain-of-interest’* genomes (a larger number of genomes might work, but has not been extensively tested). The number of *‘outgroup’ or ‘extra’* genomes may exceed this limit because these will be used for the (computationally less intensive) SLST target *in silico* PCR validation step only; nevertheless, we advise not to provide more than 500 of these additional genomes for similar reason. The input genomes can be provided as GenBank (compatible extensions are .gbk, .gbff or .gb) or as nucleotide FASTA files (extensions .fasta, .fas, .fa, .fsa, .fna or .seq are recognized), can be either single- or multi-contig (or scaffolds), and are allowed to have multiple files of the same type for each unique genome (GenBank or FASTA). The configuration file is mandatory, and has to contain all of the above information for each genome, in a tab-delimited format. A *config.txt* example file is provided in the pipeline upon download, including an example run on *Bifidobacterium* (both found in /TaxPhlAn/Pipeline-Design/). The location

(path) of the input genomes directory and of the configuration file must be provided through argument values `--indir` and `--conf` on the command line, respectively. The provided configuration file will automatically be checked on required input information and format, and provided genome files are screened for any inconsistencies in file names as listed in the configuration file. Common user-input problems such as format inconsistencies or unmet criteria will be reported, and the user will be asked to specify, change, include or remove information accordingly. Providing an output directory is optional through the `--outdir` command, if not provided, output will be directed to ./TaxPhlAn.

3.2 Pre-processing phase: Input file reformatting and annotation of genomes. (Phase I-b)

In this phase, for each input genome, its contig(s) is extracted from its genome file(s), and if applicable, multiple contigs are concatenated into a single genome file and given a unique strain identifier (Strain1, Strain2, Strain3, etc.). In this process, a log-file of contigs (or scaffolds) joined to the same genome, and a translation table linking the new genome identifiers to their original contig(s) and genome will automatically be generated into (tab-delimited or FASTA-style, respectively) flat text files. After running properly, this pre-processing phase yields the user with cleaned-up, single-file standardized input genomes (in FASTA format, .fna) required for Prokka automated genome annotation [7]. In order to speed-up calculation time, annotation can be performed on multiple processor cores simultaneously (default number of cores is 1, maximum of 16 supported). For quality control (QC) purposes, a statistics file will be generated in the main output directory (‘*statistics.txt*’), providing for each input genome information on its genome size, number of scaffolds / contigs, the GC-percentage and number of non-ATCG characters in the genome.

3.3 Orthology calculations and phylogenetic analysis. (Phase II)

For our proposed strain-typing method, it is key to determine single-copy, one-to-one orthologous gene clusters. The orthologous groups (OG) are calculated with orthAgogue [8] and requires the resulting output of an all-to-all protein BLAST (BLASTp; default settings) [9] as input data. These orthology calculations are based on a combination of the Inparanoid [10, 11] and Markov clustering

(MCL) [12] algorithms underlying orthAgogue. In our TaxPhlAn pipeline, (Python-enabled) multiprocessing is supported for BLASTp, as well as for orthAgogue and Prokka; however, no more than 16 cores will be used by Prokka as internal tests have indicated that beyond that number computation time again increases by effect of supposed hard disk drive overhead processing (data not shown). The orthology matrix is reported as a tab-separated text file (*'matrix.txt'*, or '*matrix.annotated.txt'*, in PhyloTree_OUT/ of main output directory). For additional genome analysis, all single-copy one-to-one OG are determined and used for construction of a phylogenetic tree by: (**i**) alignment of the orthologous clusters (DNA-based alignments) by MUSCLE [13] (default settings), (**ii**) concatenation of all informative single nucleotide polymorphism (SNP) positions of each OG alignment into pseudo genes (i.e. one long stretch of SNPs / pseudo gene for each genome, based on all OG alignments), and finally, (**iii**) reconstruction of a phylogenetic tree based on the pseudo genes by FastTree [14] (default settings), which generates an iTOL-compatible newick tree file [15, 16] for visualization purposes, and a subsequent text-based phylogenetic tree by the command-line tool *newicktotxt* from NJplot [17, 18].

3.4 Selection of core gene clusters for candidate marker genes. (Phase III-a)

Based on orthology calculations by orthAgogue, single-copy OG (i.e. gene clusters; arbitrarily denoted by orthAgogue as cluster_1, cluster_2, cluster_3, etc.) are selected that meet the following criteria: (**i**) do not contain paralogs or multiple genes that are derived from the same genome, and (**ii**) are shared by at least 80% of the input genomes (default value, this setting can be adjusted). Importantly, as open reading frames (ORF) as predicted by Prokka are used for OG and gene cluster calculations, only gene-coding regions are considered; and consequently, inter-genetic regions which by their nature are not (strongly) subjected to evolutionary pressure (i.e. not typically carrying phylogenetically-linked sequence patterns) are dismissed. This process results in core clusters, and is reported by the pipeline as a tab-delimited text file.

3.5 Prediction of variable regions in candidate core clusters. (Phase III-b)

In the process of variable regions (VR) prediction, the TaxPhlAn algorithm starts by identifying highly conserved regions (CR) in the predicted core clusters, by a customizable set of rules. The core clusters are aligned with MUSCLE (default settings) [13]. Based on these cluster alignments, the algorithm builds-up conserved regions from alignment start position to end. A conserved region is identified in an iterative fashion until a (penalty) limit is reached for any of the parameters as described in the next section. Hereafter, the conserved region is reported, and building of the next conserved region begins from the subsequent alignment position.

The algorithm for predicting variable regions computationally and iteratively evaluates all genetic regions within a certain range, in-between any combination of conserved regions pairs (i.e. conserved regions that have been identified based on specified settings at the first step of variable region detection). The default sequence span considered is 100-500nt, and this can be customized with the pipeline commands `--minlen` and `--maxlen`, for minimum and maximum variable region length, respectively. Note that, for this reason, one or more conserved regions can also be inside such a candidate variable region, but is nevertheless always flanked at its ends by a distinct conserved region pair suitable for SLST primer design. Consequently, any possible genetic region flanked by conserved sequences will be evaluated for its phylogenetic resolving power, unless it complies with the user-selected variable region length.

3.6 Penalizing system when searching for conserved (and variable) regions. (Phase III-c)

For finding conserved regions in cluster alignments, first, the TaxPhlAn algorithm will define a reference sequence based on the cluster alignment. For this reference sequence, the most frequent base at each alignment position is determined. In the common case of high base ambiguity on an alignment position, a degenerate IUPAC code base is allocated to the reference. A degenerate IUPAC base will be introduced to the reference sequence when 2, 3, or 4 different bases show >33%, >25%

or >20% dominance at an alignment position, respectively (listed numbers are default values, and are customizable with commands `--n2`, `--n3` and `--n4`).

After the reference (consensus) sequence has been defined, the TaxPhlAn algorithm will iteratively build-up a conserved sequence by comparing all individual cluster sequences to the reference, again based on the same cluster alignment. At each alignment position, the percentage of mismatches to the reference will be calculated, and if the reference sequence has a degenerate base at that position this will be recorded. If the percentage of mismatches is not higher than 20% (default), and if the number of degenerates in the already build-up conserved sequence does not exceed 4 (default), then the (degenerate) base at that position of the reference sequence is added to that conserved sequence (these two algorithm parameters are customizable with the pipeline commands `--mism` and `--deg`, respectively). This process is repeated at each cluster alignment position, until one of the (penalty) limits is exceeded, but also in case of IUPAC bases in any of the aligned sequences (not the reference), or when encountering alignment-introduced gaps. These unmet characteristics to further extend the already build-up conserved sequence are called 'failures' (set to 1 by default), and is customizable with command line option `--fails`.

When the maximum number of failures has been reached: (**i**) conserved sequence building stops immediately, and (**ii**) the currently build-up conserved sequence up to that alignment position is further evaluated. Thereafter, (**iii**) the conserved sequence is checked for its length, and is only reported if it meets the user-specified minimal conserved region length in order to facilitate primer design by Primer3 (15 by default, customizable with the pipeline command `--plen`) [19, 20]. Finally, (**iv**) all parameters are cleared from their penalties (i.e. reset to zero) and (**v**) conserved sequence building initiates again at the next alignment position.

3.7 in silico primer design and evaluation of variable (SLST) regions. (Phase IV)

The *in silico* predicted candidate variable regions are screened on a number of characteristics: a.o. (**i**) resolving power, (**ii**) size of the target region, (**iii**) number of genomes that contain the candidate

region, (**iv**) correlation number to full-genome-based genome phylogeny and (**v**) number of primer sets available for that region. Thereafter, SLST candidates reported in the resulting output file are sorted first on Spearman correlation to original full-genome phylogeny (maximum likelihood-based; ML), then on relative discrimination (defined as follows: (1 / (# input genomes + (# input genomes – # genomes recognized by primers of that candidate region, i.e. # hits)) * # unique VR sequences for that candidate region)). Primer sets of the top-level region candidates (100, by default) are selected and tested by PrimerProspector [21] in an *in silico* PCR on the full genome content of all input genomes (i.e. on all user-provided in- and outgroup genomes). For each primer set per candidate region, results (i.e. amplicons) from that *in silico* PCR by PrimerProspector are again analyzed on (**i**) number of amplicons identified (measure for number of genomes recognized by the primer pair), (**ii**) number of unique amplicons (in other words, resolving power of the region amplified), (**iii**) minimum and maximum amplicon size, and (**iv**) correlation value of amplicon-based phylogenetic relatedness to full-genome-based phylogeny.

Finally, top candidate regions and primers can be checked with Primer-BLAST to the NCBI's nt/nr database through a hyperlink provided by the pipeline, in order to check for undesired analogous gene regions and potential PCR-derived off-targets, respectively (found in SLST_OUT/ of the pipeline's main output directory: *Query-for-OFF-Targets-by-PrimerBLAST.html*) [22]. A final report named '*TaxPhlAn-candidates-REPORT.sorted.validated.txt'* is reported in this same directory. Alternatively, PDF files with a phylogenetic tree and alignment visualization of the top SLST candidates are generated based on their *in silico* performance, and can be found in the main output directory SLST_OUT/PPVal2-genomes/ (see for an impression Suppl. Fig. S3 for the main candidate of our *Bifidobacterium* example dataset).

4. TaxPhlAn (Module B): SLST data analysis (oligotyping) workflow (see Suppl. Fig. S2)

4.1 Data preparation phase: mandatory input and quality control. (Phase I)

An SLST oligotyping example run and test dataset is provided in the pipeline upon download (both found in /TaxPhlAn/Pipeline-Oligotyping/). To initiate, the main SLST data analysis wrapper script needs to be executed (‘*TaxPhlAn-SLST-Oligotyping-wrapper.py*’), specifying the input directory with FASTA or FASTQ files with the `--indir` option (mandatory input; reads should be assembled in case of paired-end sequencing), and optionally a user-specified output directory with the `--outdir` option. Furthermore, a mandatory reference alignment in FASTA format containing SLST reference sequences has to be supplied by the user with the `--fasta` option. This file (unique for each SLST candidate) is generated by the TaxPhlAn pipeline Module A, and can be found in SLST_OUT/PPVal2-genomes/ of the main output directory). Additional SLST references can manually be appended to the existing list (for example, references from other sources or databases, and any reference not taken along in the initial TaxPhlAn pipeline). Finally, the expected nucleotide length of the SLST sequences (i.e. alignment length, based on the references, primers included) has to be provided with the `--len` option (mandatory). Hereafter, reads are filtered on this expected length, only allowing reads within a three nucleotide (shorter) length of the reference SLST alignment to pass the filter. The read numbers for each sample are reported in an intermediate read statistics report on QC numbers and filtering: *‘1_QC-report.txt’* in the main output directory.

4.2 Oligotyping by allele building. (Phase II)

Following the intermediate QC step, the pipeline run will be paused, thereby allowing for user-guided (manual) sample deselection for (suspected) spurious and / or low quality samples. This short user-interaction step will prevent use of erroneous SLST sequences for oligotyping; deselected samples are excluded in any further analysis. Only SLST reads passing above-described sequence length filter are aligned to the reference alignment by PyNAST, applying default settings [23]; reads longer than the reference alignment are discarded as they disrupt the reference alignment, which is not allowed by

the PyNAST algorithm. Reads that are mapped by PyNAST to the reference alignment are retained for further analysis and appended to the existing reference alignment, thereby building an SLST reads super-alignment. In order to assign the informative SNP positions in the SLST sequences we determined the Shannon diversity index (SDI) for each position in the SLST super-alignment, by adopting the Python module Shannon.py for calculating Shannon entropy (as published at GitHubGist: `https://gist.github.com/audy/783125`). Different SDI thresholds are evaluated (0.1 to 0.9, with steps of 0.1), and the number of informative SNP positions is calculated for each threshold, together with the corresponding number of unique alleles in the complete dataset. An 'allele' consists of the nucleotides present at the informative positions in a particular sequencing read. Note that output for a given SDI threshold is reported only if the number of obtained SNP positions is larger than 10; hence, this is similar to the length of the alleles (10 is the default value, this is customizable with command line argument *--minSNP*). We furthermore provide the option to exclude samples from these SDI calculations, by providing a list of samples with the *--list* option to be excluded for SDI calculations, but allele compositions of these samples will still be determined and reported. This can be relevant for positive- and negative control samples, or for instance when you have an imbalance in the number of (replication) samples for your individuals (hence, more replication samples of individual A compared to individual B could bias the SDI calculations towards individual A). Final output of this analysis phase is a list of all unique alleles found in the *entire dataset* for each SDI threshold, their overall relative abundances, and their corresponding reference genomes (if available); see flat-text file '*allele_info_complete_dataset-Th-0.*.txt*' in directory 5_Oligotyping_results/ (in tab-separated table format; sorted on the on average most abundant alleles). In the current study we used SDI thresholds of 0.6 for genus-level, and 0.2 for species-level, thresholds were empirically chosen based on work presented in this manuscript.

4.3 SLST sequences allele matching and scoring. (Phase III-a)

Each individual SLST read of the samples to be analyzed is converted to an allele depending on the different SDI threshold thresholds, and classified based on the known references, or classified as a (novel) unknown taxonomical entity (but with known phylogenetic placement). Note that this classification is performed without allowing mismatches in the allele sequences, hence only perfect hits are allowed.

4.4 Phylogenetic analysis of alleles, and data visualization. (Phase III-b)

For data output at each SDI threshold: annotation, data and phylogenetic tree files for use with iTOL [16] are automatically generated to accommodate the user with visualization of their results. In short, an allele-based phylogenetic tree figure of the top *N* most abundant alleles is generated as a PDF file, combined with a Bray-Curtis clustered heatmap of all SLST samples (underlying log10-transformed SLST relative abundances data). The individual iTOL-compatible files can be found in the directory 5_Oligotyping-results (and see 6_iTOL/ for intermediate files used for communication with the iTOL API at `https://itol.embl.de/help.cgi#batch`). Final visualization results for each SDI threshold are reported as '*iTOL-results-SDI0.*.pdf*' in the main output directory.

4.5 Oligotyping data results and reporting. (Phase IV)

In the final analysis phase, the number of reads corresponding to each unique allele is counted for every selected sample in the input directory, and is reported as an allele-to-sample (i.e. microbiota-to-sample, in those cases where an allele corresponds to a known reference) compositional matrix. Final output of these alleles *per sample* for each SDI threshold can be found in the directory 5_Oligotyping_results/ as flat-text files (in tab-separated table format) including allele absolute or relative abundances and taxonomical classifications: '*allele_Counts_per_Sample-Th-0.*.txt*' for absolute, and '*allele_RelAbund_per_Sample-Th-0.*.txt*' for relative abundances (sorted on the dataset-wide on average most abundant alleles). FASTA files with the *N* most abundant alleles in the dataset

for each SDI threshold (‘*alleles-selected-topN-Th-0.*.fasta*’) and the newick files thereof (‘*alleles-selected-topN-Th-0.*.newick*’) can also be found in the directory 5_Oligotyping_results/. This top *N* is set to 100 by default, this value is customizable with `--topN` command-line argument. In addition, iTOL-compatible newick and annotation files to generate phylogenetic SLST trees (including allele alignments and heatmaps of SLST abundances, etc.) can be found in the same directory. A final summary report is provided in the main working directory, providing the overall (and for each SDI threshold threshold) SLST read statistics: ‘*5_Summary-of-Results.txt*’. Furthermore, this report summarizes relevant information, a.o. with regard to number of informative SNP positions, number of alleles detected, number of reference genomes identified, and the dataset-wide overall relative abundance of the top *N* alleles.

5. TaxPhlAn pipeline dependencies : Software

Software, third party open source Programs and Tools, required to run TaxPhlAn :

**name**	**version**	**reference**
ARAGORN	1.2.38	[24]
BioPerl	1.7.2	[25]
BLAST+ *(blastn, blastp, makeblastdb)*	2.2.31+	[9]
CPAN	2.11	[26]
EMBOSS (*distmat, needle*)	6.6.0.0	[4]
FastTree (MP)	2.1.10	[14, 27]
FASTX Toolkit *(fastq_to_fasta)*	0.0.14	[28]
GNU Parallel	20180622	[29]
HMMER	3.1	[30, 31]
Infernal	1.1.2	[32]
Inparanoid	4.1	[10, 11]
iTOL 4 *(requires internet connection)*	web	[15, 16, 33, 34]
MCL	14-137	[12]
MUSCLE	3.8.31	[13]
Newick Utilities *(nw_reroot)*	1.6	[35]
newicktotxt	2.4.7	[17]
NumPy	1.14.5	[36, 37]
orthAgogue	1.0.1	[8]
PEAR	0.9.11	[38]
Perl	5.22.1	[39]
PerlBrew	0.84	[40]
PHYLIP *(neighbor)*	3.6	[41, 42]
Primer3	2.3.7	[19, 20, 43]
Primer-BLAST	web	[22]
PrimerProspector	1.01	[21]
Prodigal	2.6.3	[44]
Prokka	1.14	[7]
PyCogent	1.9	[45]
PyNAST	0.1	[23]
Python	2.7.15	[46]
R	3.3.3	[47]
SciPy	1.1.0	[48, 49]
tbl2asn	25.6	[50]
UCLUST	1.2.22	[5]

6. TaxPhlAn pipeline dependencies : Libraries

Python Libraries :

argparse
argv
collections
datetime
dateutil
dendropy
ete2
getopt
glob
inspect
math
matplotlib
multiprocessing
numpy
operator
os
pyparsing
random
re
scipy
scripts.shannon
scripts.textformatting
shlex
shutil
signal
string
stringio
subprocess
sys
time
traceback

Perl Libraries :

Bio::DB::Fasta
Bio::Perl
Bio::Seq
File::Basename
File::Spec::Functions
List::Util
Module::Build

References of Supplementary Methods

1. Kitts, P.A., et al., *Assembly: a resource for assembled genomes at NCBI.* Nucleic Acids Res, 2016. 44(D1): p. D73-80.
2. Altschul, S.F., et al., *Basic local alignment search tool.* J Mol Biol, 1990. 215(3): p. 403-10.
3. Pruesse, E., et al., *SILVA: a comprehensive online resource for quality checked and aligned ribosomal RNA sequence data compatible with ARB.* Nucleic Acids Research, 2007. 35(21): p. 7188-7196.
4. Rice, P., I. Longden, and A. Bleasby, *EMBOSS: the European Molecular Biology Open Software Suite.* Trends Genet, 2000. 16(6): p. 276-7.
5. Edgar, R.C., *Search and clustering orders of magnitude faster than BLAST.* Bioinformatics, 2010. 26(19): p. 2460-1.
6. website. *FASTQC bioinformatics tool*. Available from: http://www.bioinformatics.babraham.ac.uk/projects/fastqc/.
7. Seemann, T., *Prokka: rapid prokaryotic genome annotation.* Bioinformatics, 2014. 30(14): p. 2068-9.
8. Ekseth, O.K., M. Kuiper, and V. Mironov, *orthAgogue: an agile tool for the rapid prediction of orthology relations.* Bioinformatics, 2014. 30(5): p. 734-6.
9. Camacho, C., et al., *BLAST+: architecture and applications.* BMC Bioinformatics, 2009. 10: p. 421-421.
10. Remm, M., C.E. Storm, and E.L. Sonnhammer, *Automatic clustering of orthologs and in-paralogs from pairwise species comparisons.* J Mol Biol, 2001. 314(5): p. 1041-52.
11. O'Brien, K.P., M. Remm, and E.L. Sonnhammer, *Inparanoid: a comprehensive database of eukaryotic orthologs.* Nucleic Acids Res, 2005. 33(Database issue): p. D476-80.
12. Enright, A.J., S. Van Dongen, and C.A. Ouzounis, *An efficient algorithm for large-scale detection of protein families.* Nucleic Acids Res, 2002. 30(7): p. 1575-84.
13. Edgar, R.C., *MUSCLE: multiple sequence alignment with high accuracy and high throughput.* Nucleic Acids Res, 2004. 32(5): p. 1792-7.
14. Price, M.N., P.S. Dehal, and A.P. Arkin, *FastTree: computing large minimum evolution trees with profiles instead of a distance matrix.* Mol Biol Evol, 2009. 26(7): p. 1641-50.
15. website. *iTOL (interactive Tree of Life) bioinformatics tool*. Available from: http://itol.embl.de/.
16. Letunic, I. and P. Bork, *Interactive tree of life (iTOL) v3: an online tool for the display and annotation of phylogenetic and other trees.* Nucleic Acids Res, 2016. 44(W1): p. W242-5.
17. website. *NJplot (newicktotxt) bioinformatics tool*. Available from: http://doua.prabi.fr/software/njplot.
18. Perrière, G. and M. Gouy, *WWW-query: An on-line retrieval system for biological sequence banks.* Biochimie, 1996. 78(5): p. 364-369.
19. Koressaar, T. and M. Remm, *Enhancements and modifications of primer design program Primer3.* Bioinformatics, 2007. 23(10): p. 1289-1291.
20. Untergasser, A., et al., *Primer3—new capabilities and interfaces.* Nucleic Acids Research, 2012. 40(15): p. e115-e115.
21. Walters, W.A., et al., *PrimerProspector: de novo design and taxonomic analysis of barcoded polymerase chain reaction primers.* Bioinformatics, 2011. 27(8): p. 1159-61.
22. Ye, J., et al., *Primer-BLAST: a tool to design target-specific primers for polymerase chain reaction.* BMC Bioinformatics, 2012. 13: p. 134.
23. Caporaso, J.G., et al., *PyNAST: a flexible tool for aligning sequences to a template alignment.* Bioinformatics, 2010. 26(2): p. 266-7.
24. Laslett, D. and B. Canback, *ARAGORN, a program to detect tRNA genes and tmRNA genes in nucleotide sequences.* Nucleic Acids Research, 2004. 32(1): p. 11-16.

25. Stajich, J.E., et al., *The Bioperl toolkit: Perl modules for the life sciences.* Genome Res, 2002. 12(10): p. 1611-8.
26. website. *CPAN, The Comprehensive Perl Archive Network*. Available from: http://www.cpan.org/.
27. Price, M.N., P.S. Dehal, and A.P. Arkin, *FastTree 2--approximately maximum-likelihood trees for large alignments.* PLoS One, 2010. 5(3): p. e9490.
28. website. *FastX-Toolkit bioinformatics tool*. Available from: http://hannonlab.cshl.edu/fastx_toolkit/.
29. website. *GNU parallel - a shell tool for executing jobs in parallel using one or more computers*. Available from: http://www.gnu.org/software/parallel/.
30. website. *HMMER: biosequence analysis using profile hidden Markov models*. Available from: http://hmmer.org/.
31. Eddy, S.R., *Multiple alignment using hidden Markov models.* Proc Int Conf Intell Syst Mol Biol, 1995. 3: p. 114-20.
32. Nawrocki, E.P. and S.R. Eddy, *Infernal 1.1: 100-fold faster RNA homology searches.* Bioinformatics, 2013. 29(22): p. 2933-5.
33. Letunic, I. and P. Bork, *Interactive Tree Of Life (iTOL): an online tool for phylogenetic tree display and annotation.* Bioinformatics, 2007. 23(1): p. 127-8.
34. Letunic, I. and P. Bork, *Interactive Tree Of Life v2: online annotation and display of phylogenetic trees made easy.* Nucleic Acids Res, 2011. 39(Web Server issue): p. W475-8.
35. Junier, T. and E.M. Zdobnov, *The Newick utilities: high-throughput phylogenetic tree processing in the UNIX shell.* Bioinformatics, 2010. 26(13): p. 1669-70.
36. website. *NumPy - for scientific computing with Python*. Available from: http://www.numpy.org/.
37. Oliphant, T.E., *Guide to NumPy.* USA: Trelgol Publishing, 2006.
38. Zhang, J., et al., *PEAR: a fast and accurate Illumina Paired-End reAd mergeR.* Bioinformatics, 2014. 30(5): p. 614-20.
39. website. *The Perl Programming Language*. Available from: http://www.perl.org/.
40. website. *PerlBrew, an admin-free Perl installation management tool*. Available from: http://perlbrew.pl/.
41. website. *PHYLIP - a bioinformatics package of programs for inferring phylogenies*. Available from: http://evolution.genetics.washington.edu/phylip.html.
42. Baum, B.R., *PHYLIP: Phylogeny Inference Package. Version 3.2. Joel Felsenstein.* The Quarterly Review of Biology, 1989. 64(4): p. 539-541.
43. website. *Primer3 - primer design program*. Available from: http://primer3.ut.ee/.
44. Hyatt, D., et al., *Prodigal: prokaryotic gene recognition and translation initiation site identification.* BMC Bioinformatics, 2010. 11: p. 119.
45. Knight, R., et al., *PyCogent: a toolkit for making sense from sequence.* Genome Biol, 2007. 8(8): p. R171.
46. website. *The Python Programming Language*. Available from: http://www.python.org/.
47. website. *R: The R Project for Statistical Computing*. Available from: http://www.r-project.org/.
48. website. *SciPy - a Python-based ecosystem of open-source software for mathematics, science, and engineering.*; Available from: http://www.scipy.org/.
49. Oliphant, T.E., *Python for Scientific Computing.* Comput. Sci. Eng., 2007. 9:10–20.
50. website. *tbl2asn - program that automates the creation of sequence records for submission to GenBank*. Available from: http://www.ncbi.nlm.nih.gov/genbank/tbl2asn2/.

Supplementary Data

A generic workflow for Single Locus Sequence Typing (SLST) design and subspecies characterization of microbiota.

Thomas H.A. Ederveen 1-3 #, Jos P.H. Smits 2, Karima Hajo 1, Saskia van Schalkwijk 3, Tessa A. Kouwenhoven 2, Sabina Lukovac 3, Michiel Wels 1,3, Ellen H. van den Bogaard 2, Joost Schalkwijk 2, Jos Boekhorst 1,3, Patrick L.J.M. Zeeuwen 2 and Sacha A.F.T. van Hijum 1,3

1. Center for Molecular and Biomolecular Informatics (CMBI), Radboud Institute for Molecular Life Sciences (RIMLS), Radboud University Medical Center (Radboudumc), Nijmegen, The Netherlands.

2. Department of Dermatology, RIMLS, Radboudumc, Nijmegen, The Netherlands.

3. NIZO, Ede, The Netherlands.

# Corresponding author: Thomas H.A. Ederveen, Center for Molecular and Biomolecular Informatics (CMBI), Radboud Institute for Molecular Life Sciences (RIMLS), Radboud University Medical Center (Radboudumc), Geert Grooteplein Zuid 28 (route 260), 6525 GA, Nijmegen, The Netherlands. E: Tom.Ederveen@radboudumc.nl, T: 0031-243619390

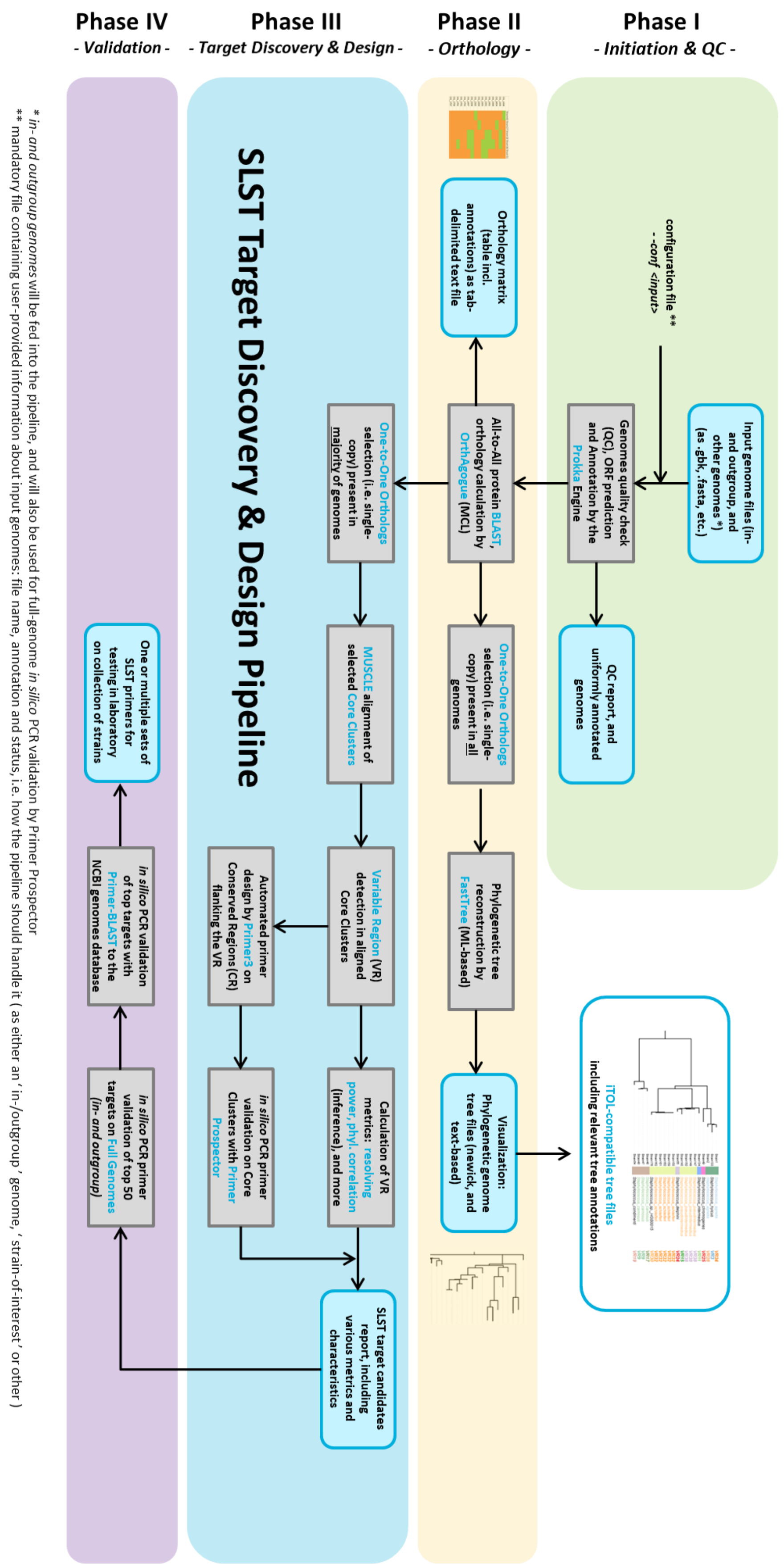

Suppl. Figure S1. Overview of TaxPhlAn SLST Discovery and Design pipeline steps (Module A).

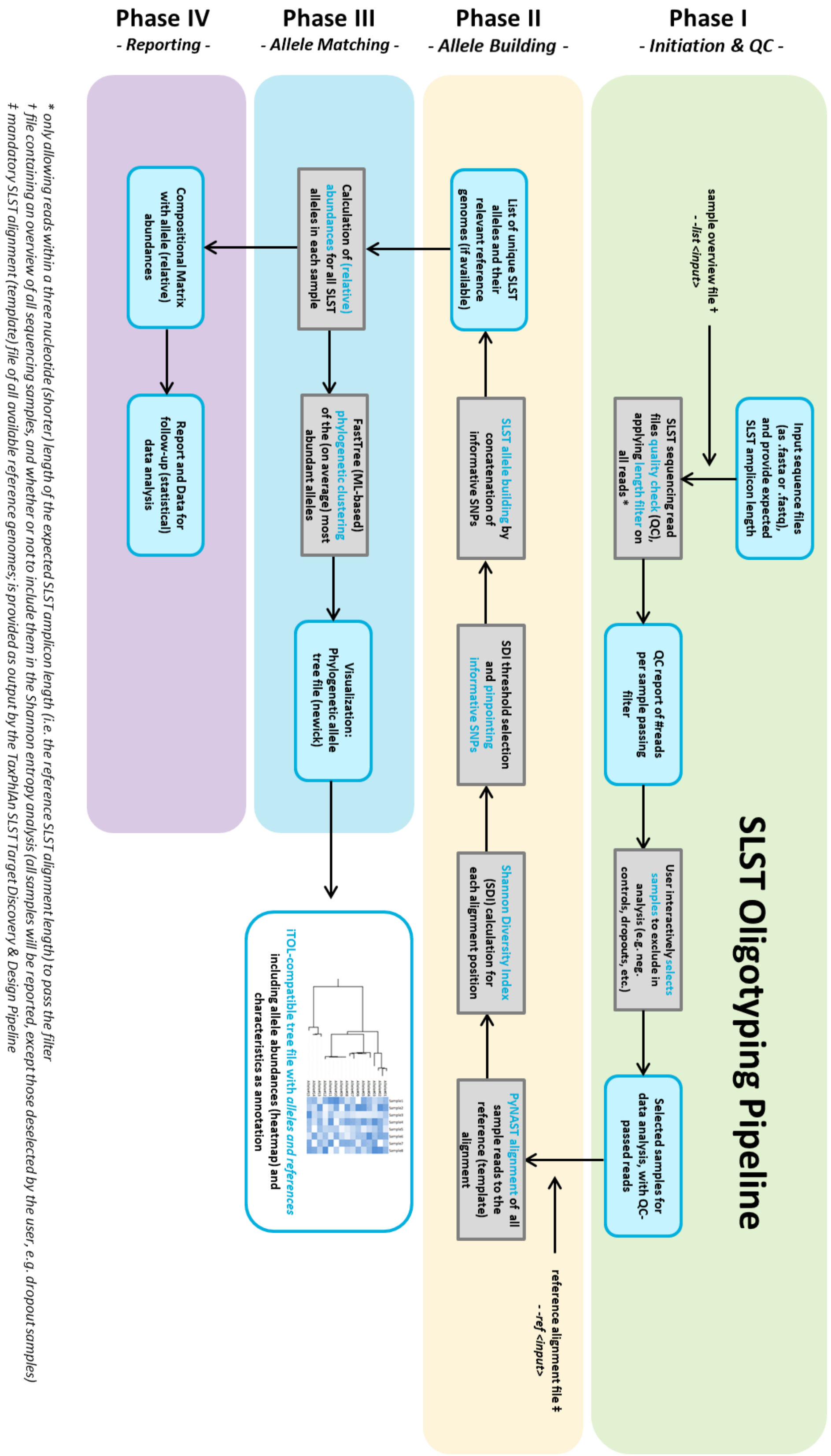

Suppl. Figure S2. Overview of TaxPhlAn SLST Oligotyping pipeline steps (Module B).

Suppl. Figures S3-S6. SLST candidates as predicted by TaxPhlAn correlates with the actual phylogenetic distances between genomes.

Shown in the PDF files (listed on the next page) is for each benchmark genome dataset a phylogenetic tree based on concatenated single nucleotide polymorphism (SNP) positions in the aligned core genome (one-to-one single-copy orthologous genes) of all genomes in that benchmark dataset (see Table 1 and Suppl. Tables S1-S4 for additional information on the benchmark datasets). One can appreciate that different variable regions (VR) of the SLST targets cluster almost entirely with the different bacterial clades based on full-genome information, and this was observed for all four bacterial datasets. Corresponding Spearman correlation values were on average 0.775 rho, and alternatively, Pearson's r correlation values were comparable (Suppl. Tables S6-S9). Other sequence distance methods such as DISTMAT [1] provided similar outcomes (Suppl. Tables S6-S9). Altogether, for automated SLST target prioritizing we decided to adopt ML-based Spearman correlations as we believe this is best practice, and data shows little difference from DISTMAT-based distances or correlation by Spearman. The phylogenetic trees are built with FastTree [2] which is maximum likelihood-based, and visualized with iTOL [3]. For the top SLST candidate of each benchmark genome dataset as analyzed with TaxPhlAn (Suppl. Tables S6-S9), the SLST variable regions (VR) are indicated with different colors and numbers (e.g. VR10), and the first 100 nucleotides (nt) of the sequence alignment of the SLST regions is plotted on the right (aligned by MUSCLE [4]). The number followed by an asterisk in between the brackets, such as (002*), represents the number of times that unique VR was found in the dataset. Blanc line means that based on the *in silico* PCR analysis by PrimerPropsector [5] no primer hit was available for that genome.

Files :
S3 Bifidobacterium-SLST-ID-630.24-34.21-21.iTOL.pdf
S4 Escherichia-Shigella-SLST-ID-1978.3-8.32-32.iTOL.pdf
S5 Propionibacterium-SLST-ID-1414.3-7.9-9.iTOL.pdf
S6 Staphylococus-SLST-ID-1238.2-13.3-3.iTOL.pdf

Suppl. Figure S7. ***Staphylococcus*** **full-genome phylogenetic tree projected with 16S clusters and SLST OG #1123 variable regions.**

Shown in the PDF file is for the *Staphylococcus* dataset a phylogenetic tree based on concatenated single nucleotide polymorphism positions in the aligned core genome of all genomes in that benchmark dataset (n = 200). The phylogenetic tree is built with FastTree [2] which is maximum likelihood-based, and visualized with iTOL [3]. SLST candidate OG #1123 variable regions (VR) are indicated with different colors and numbers (e.g. VR10). In total, we identified 48 unique variable regions for OG #1123 (see Suppl. Table S10). If no VR was indicated (blanc line) this means that based on the *in silico* PCR analysis by PrimerPropsector [5] no primer hit was available for that genome (is only the case for Strain155). Colored blocks on the right of the phylogenetic tree represent unique 16S clusters based on full-length 16S sequences from these *Staphylococcus* genomes clustered on 99.7% identity (see '*Selection of representative Staphylococcus genomes for TaxPhlAn target discovery*' in Methods for more details).

File :
S7 SLST-Staphylococcus-OG1123.pdf

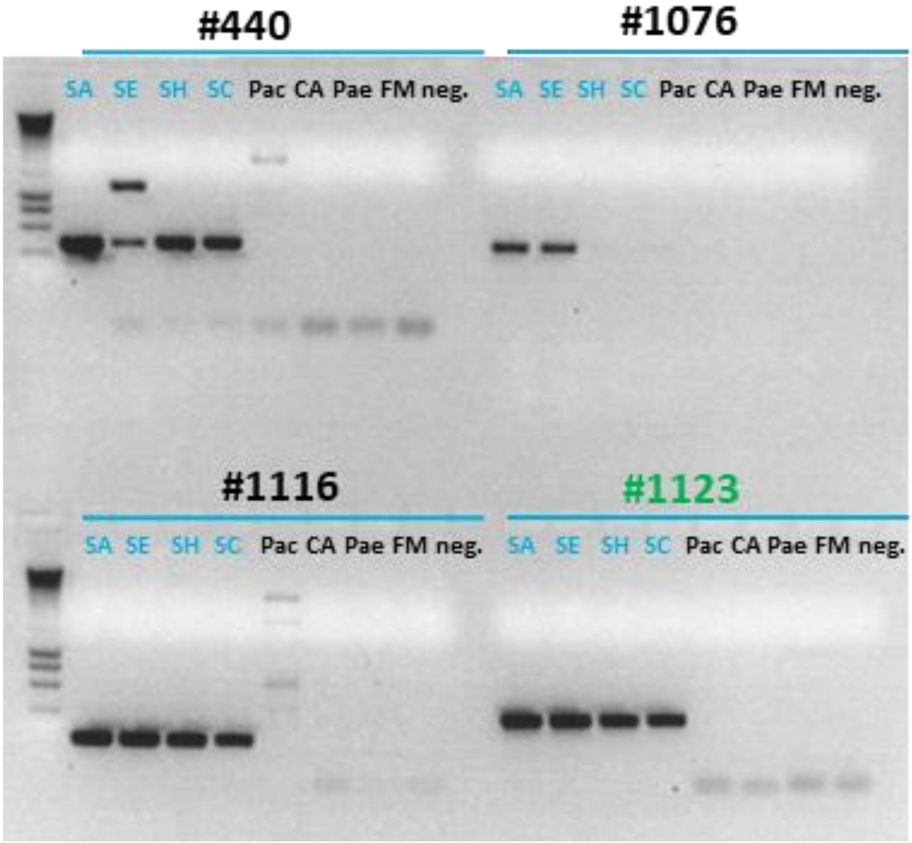

Suppl. Figure S8. PCR validation of SLST candidates primer sets with *Staphylococcus* species and common skin commensals.

SLST candidates (see Supplementary Table S13) OG #1116 and #440 showed cross-reactivity with *P. acnes*, in addition #440 had an off-target for *S. epidermidis*. OG # 1076 did not recognize *S. hominis* and S*. capitis*. In conclusion, OG #1123 was the most suitable candidate here with recognition of all Staphylococci and without cross-reactivity with common skin commensals. DNA ladder used: 200bp. Note that the lowest slightly visible bands are primer-dimers. SA: *S. aureus*, SE: *S. epidermidis*, SH: *S. hominis*, SC: *S. capitis*, Pac: *Propionibacterium acnes*, CA: *Corynebacterium aurimucosum*, Pae: *Pseudomonas aeruginosa*, FM: *Finegoldia magna*, neg: negative control with sterile water. The SLST OG #1123 (dark green) is our final SLST target which survived all *in silico* and lab validations, and with which our study was performed. OG #1123 is predicted to encode for 30S ribosomal protein S11.

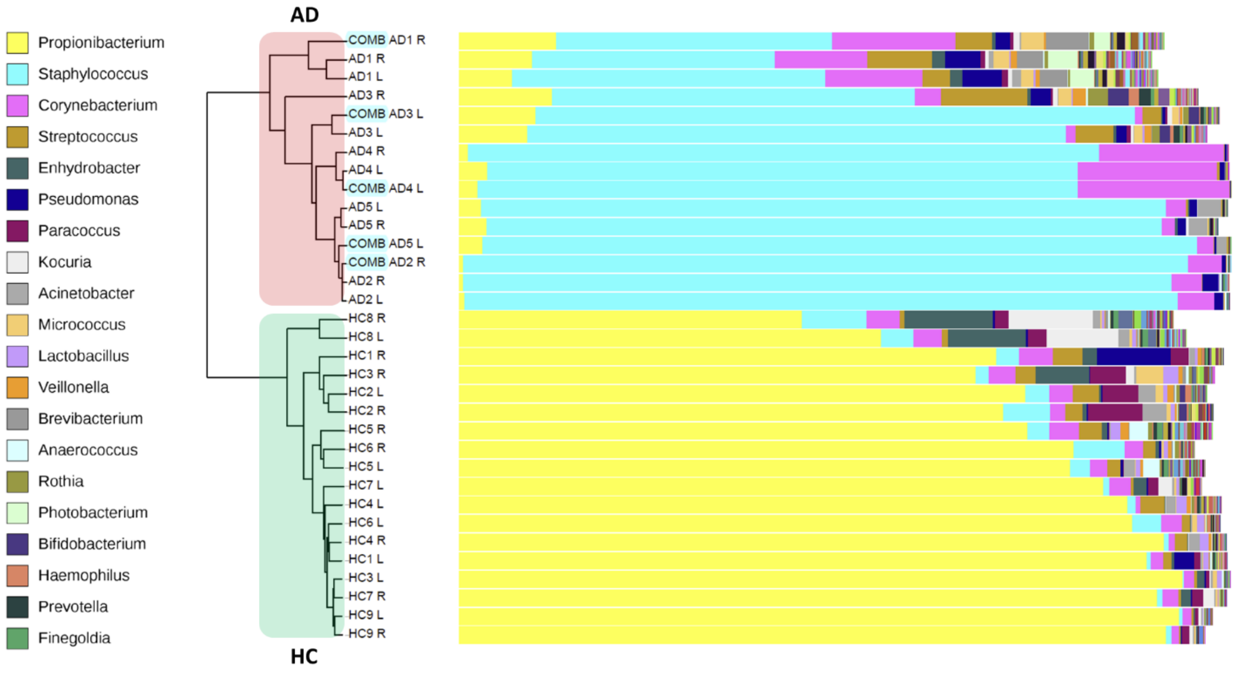

Suppl. Figure S9. Skin microbiota composition in healthy controls and AD patients: *Staphylococcus* dominates skin of AD patients at the expense of *Propionibacterium*.

Each leaf of the tree represents a single sample. Samples were clustered based on beta diversity ("between-sample distance") using weighted UniFrac as a distance measure and hierarchical UPGMA as a clustering method. Vertical bars show the relative abundance microbiota composition on the genus level (reads that could not be classified up to this level are in white), although for clustering the full microbiome information was used. The 20 most dominant genera are shown in the legend, and are sorted from high to low. Colored sample labels represent sample classes: atopic dermatitis (AD) patients in red and healthy control (HC) volunteers in green. In addition, samples marked in blue (COMB) are technical replicated for which 16S amplicons were mixed with SLST amplicons of the same sample. The figure was generated with the interactive tree of life (iTOL) program [3].

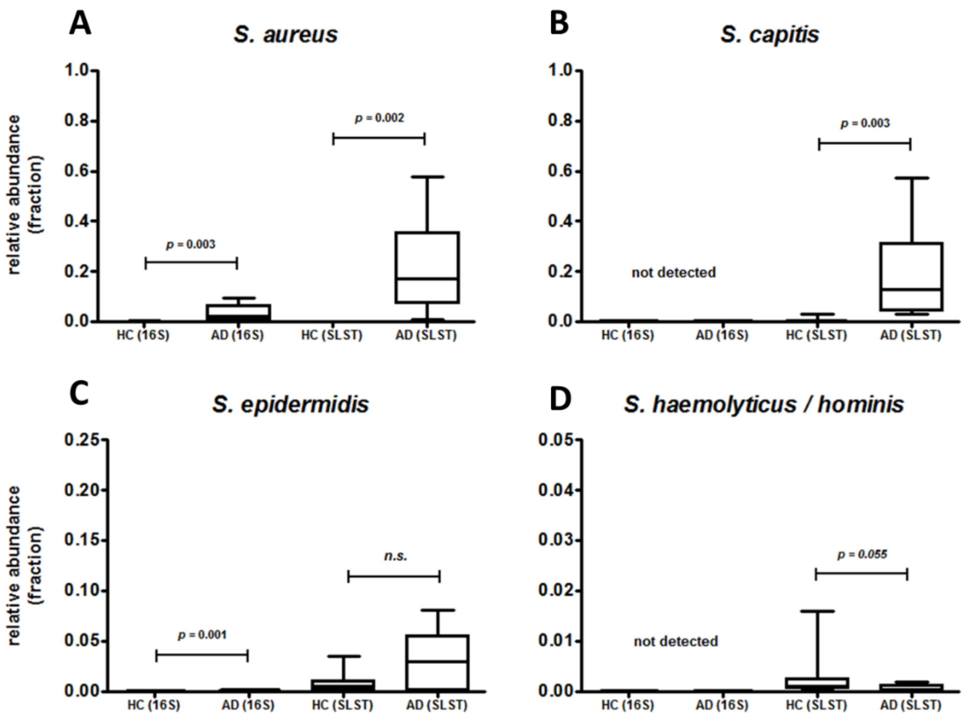

Suppl. Figure S10. ***Staphylococcus* species are increased in AD in comparison to HC.**

Plotted in bar-graphs are the relative abundance levels of *Staphylococcus* species (**A**) *S. aureus*, (**B**) *S. capitis*, (**C**) *S. epidermidis* and (**D**) *S. haemolyticus / hominis*, as measured by 16S or SLST. AD = atopic dermatitis. HC = healthy control. We mainly identified taxa of *S. aureus* and *S. capitis*, which were significantly increased in AD, in comparison to taxa of *S. epidermidis* and a cluster of *S. haemolyticus / hominis*, which were associated with healthy skin. Please note that *S. capitis* has not previously been found associated with AD, these findings should be taken with caution given our limited sample size and lack of extensive validations. The species-level SLST relative abundances plotted here have, for each sample, been corrected for the corresponding relative abundance of *Staphylococcus* (genus-level) for that sample based on 16S. Boxplots as median with interquartile range and whiskers from min to max.

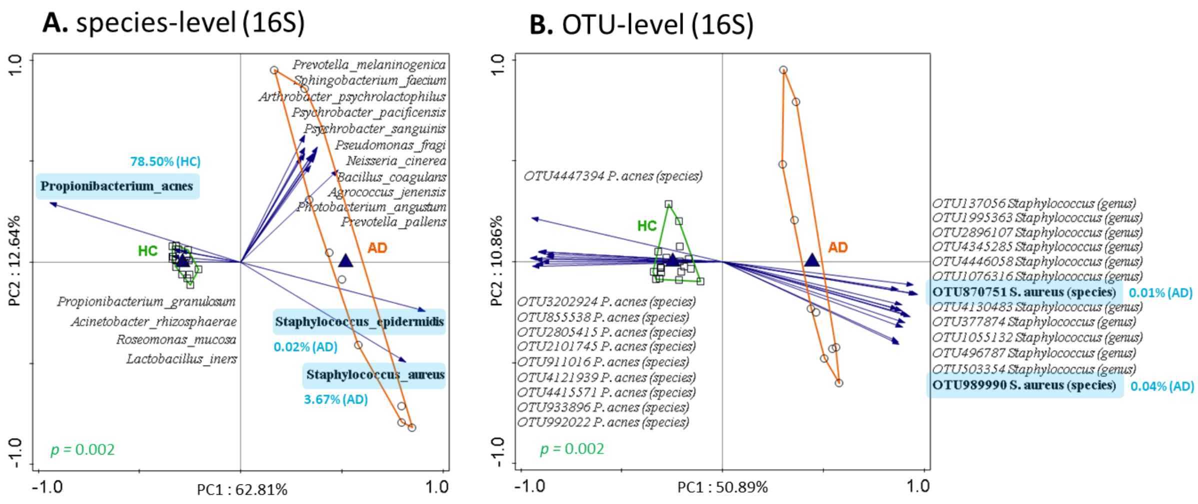

Suppl. Figure S11. Strong under-assignment of 16S *Staphylococcus* taxa below the level of genus.

The figures show a redundancy analysis (RDA) biplot of 16S data on (**A**) species-level and (**B**) OTU-level. Triangles are the centroids of the study sample groups: HC (green) and AD (orange). The blue arrows are the best-fitting bacterial taxa (names in italic or bold) with a minimum of 7.5% (A) or 75% (B) horizontal fit value, i.e. taxa that best explain the differences between the sample groups. The horizontal axis maximizes the variation in sample groups (in contrast to a principal component analysis plot, where the variation between individual samples is maximized). In RDA, samples (also) separated in the vertical direction indicates that this separation is (also) driven by other factors than the primary contrast, such as by individuality. The difference in microbiota is significant on both taxonomical level (according to a permutation test, which was corrected for individual; p = 0.002). Blue percentage numbers represent the on average relative abundance of that response variable for HC or AD; this is to show the relatively low relevance of *S. aureus*- and *S. epidermidis*-assigned genera or OTU in separation between HC and AD based on 16S data alone. SA = *S. aureus*-like taxon (allele). SC = *S. capitis*-like taxon (allele).

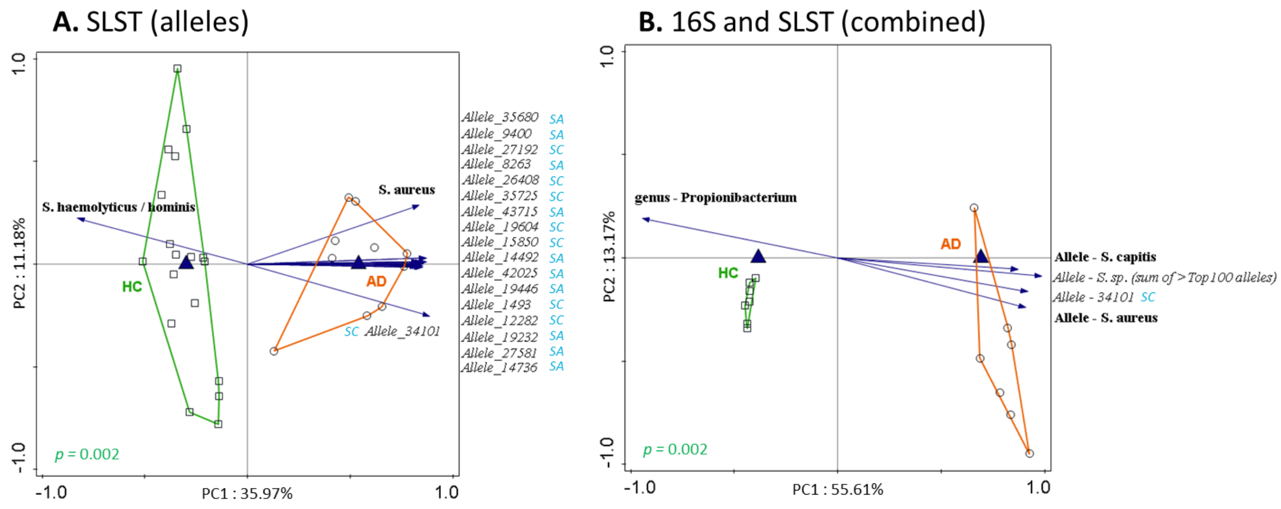

Suppl. Figure S12. The below genus-level bacterial entities discriminating HC and AD individuals is mainly explained by *S. aureus* and to a lesser extent by *S. capitis* species.

The figures show a redundancy analysis (RDA) biplot of (**A**) *Staphylococcus* SLST allele data, and of (**B**) 16S data combined with *Staphylococcus* SLST allele information. In other words, SLST data in (B) has been appended (and corrected) with available 16S data. Triangles are the centroids of the study sample groups: HC (green) and AD (orange). The blue arrows are the best-fitting bacterial taxa (names in italic or bold) with a minimum of 68% horizontal fit value, i.e. taxa that best explain the differences between the sample groups. The horizontal axis maximizes the variation in sample groups (in contrast to a principal component analysis plot, where the variation between individual samples is maximized). In RDA, samples (also) separated in the vertical direction indicates that this separation is (also) driven by other factors than the primary contrast, such as by individuality. The difference in microbiota is significant with both 16S uncorrected (A) and 16S corrected (B) data (according to a permutation test, which was corrected for individual; $p = 0.002$). Please note that *S. capitis* has not previously been found associated with AD, these findings should be taken with caution given our limited sample size and lack of extensive validations. SA = *S. aureus*-like taxon (allele). SC = *S. capitis*-like taxon (allele).

Suppl. Figure S13: Primers for *S. capitis* orthologous gene sodA are specific and sequences are conserved (*in silico* analysis).

We performed an *in silico* PCR with Primer Prospector on all *Staphylococcus* genomes in our dataset (n = 200; see Tables 1 and S4). The primers ScapF/ScapR from Iwase *et al.,* 2007 [6] generated amplicons for *S. capitis* species only and sequences were 100% identical (length = 208nt, including primers). Based on this *in silico* analysis we conclude that primers are specific and can be used for further *S. capitis* screening by qPCR. Note that *Staphylococcus-aureus-245-SAUR_41*, *sp.-TE8_1* and *warneri-562-SWAR_20* are in fact *S. capitis* strains but are misclassified in the existing databases (see Figure S7 for the *S. capitis* cluster based on core genome information; also, all genomes from this *S. capitis* cluster were recognized the primers).

CLUSTAL O (1.2.4) multiple sequence alignment of S. capitis sodA

```
Staphylococcus-capitis-SK14_3                      GATATTCAAACTGCAGTACGTAATAATGGTGGCGGTCACTTAAACCACTCATTATTCTGG  60
Staphylococcus-capitis-CR05_1                      GATATTCAAACTGCAGTACGTAATAATGGTGGCGGTCACTTAAACCACTCATTATTCTGG  60
Staphylococcus-capitis-LNZR-1_66                   GATATTCAAACTGCAGTACGTAATAATGGTGGCGGTCACTTAAACCACTCATTATTCTGG  60
Staphylococcus-capitis-CR09_12                     GATATTCAAACTGCAGTACGTAATAATGGTGGCGGTCACTTAAACCACTCATTATTCTGG  60
Staphylococcus-capitis-subsp.-capitis-AYP1020_1    GATATTCAAACTGCAGTACGTAATAATGGTGGCGGTCACTTAAACCACTCATTATTCTGG  60
Staphylococcus-capitis-CR01_1                      GATATTCAAACTGCAGTACGTAATAATGGTGGCGGTCACTTAAACCACTCATTATTCTGG  60
Staphylococcus-aureus-245-SAUR_41                  GATATTCAAACTGCAGTACGTAATAATGGTGGCGGTCACTTAAACCACTCATTATTCTGG  60
Staphylococcus-sp.-TE8_1                           GATATTCAAACTGCAGTACGTAATAATGGTGGCGGTCACTTAAACCACTCATTATTCTGG  60
Staphylococcus-capitis-VCU116_38                   GATATTCAAACTGCAGTACGTAATAATGGTGGCGGTCACTTAAACCACTCATTATTCTGG  60
Staphylococcus-warneri-562-SWAR_20                 GATATTCAAACTGCAGTACGTAATAATGGTGGCGGTCACTTAAACCACTCATTATTCTGG  60
                                                   ************************************************************

Staphylococcus-capitis-SK14_3                      GAATTATTATCACCAAATTCTGAAGAAAAAGGTGAAGTAGTAGACAAAATTAAAGAACAA  120
Staphylococcus-capitis-CR05_1                      GAATTATTATCACCAAATTCTGAAGAAAAAGGTGAAGTAGTAGACAAAATTAAAGAACAA  120
Staphylococcus-capitis-LNZR-1_66                   GAATTATTATCACCAAATTCTGAAGAAAAAGGTGAAGTAGTAGACAAAATTAAAGAACAA  120
Staphylococcus-capitis-CR09_12                     GAATTATTATCACCAAATTCTGAAGAAAAAGGTGAAGTAGTAGACAAAATTAAAGAACAA  120
Staphylococcus-capitis-subsp.-capitis-AYP1020_1    GAATTATTATCACCAAATTCTGAAGAAAAAGGTGAAGTAGTAGACAAAATTAAAGAACAA  120
Staphylococcus-capitis-CR01_1                      GAATTATTATCACCAAATTCTGAAGAAAAAGGTGAAGTAGTAGACAAAATTAAAGAACAA  120
Staphylococcus-aureus-245-SAUR_41                  GAATTATTATCACCAAATTCTGAAGAAAAAGGTGAAGTAGTAGACAAAATTAAAGAACAA  120
Staphylococcus-sp.-TE8_1                           GAATTATTATCACCAAATTCTGAAGAAAAAGGTGAAGTAGTAGACAAAATTAAAGAACAA  120
Staphylococcus-capitis-VCU116_38                   GAATTATTATCACCAAATTCTGAAGAAAAAGGTGAAGTAGTAGACAAAATTAAAGAACAA  120
Staphylococcus-warneri-562-SWAR_20                 GAATTATTATCACCAAATTCTGAAGAAAAAGGTGAAGTAGTAGACAAAATTAAAGAACAA  120
                                                   ************************************************************

Staphylococcus-capitis-SK14_3                      TGGGGTTCTTTAGATGAATTCAAAAAAGAATTTGCAGATAAAGCTGC     167
Staphylococcus-capitis-CR05_1                      TGGGGTTCTTTAGATGAATTCAAAAAAGAATTTGCAGATAAAGCTGC     167
Staphylococcus-capitis-LNZR-1_66                   TGGGGTTCTTTAGATGAATTCAAAAAAGAATTTGCAGATAAAGCTGC     167
Staphylococcus-capitis-CR09_12                     TGGGGTTCTTTAGATGAATTCAAAAAAGAATTTGCAGATAAAGCTGC     167
Staphylococcus-capitis-subsp.-capitis-AYP1020_1    TGGGGTTCTTTAGATGAATTCAAAAAAGAATTTGCAGATAAAGCTGC     167
Staphylococcus-capitis-CR01_1                      TGGGGTTCTTTAGATGAATTCAAAAAAGAATTTGCAGATAAAGCTGC     167
Staphylococcus-aureus-245-SAUR_41                  TGGGGTTCTTTAGATGAATTCAAAAAAGAATTTGCAGATAAAGCTGC     167
Staphylococcus-sp.-TE8_1                           TGGGGTTCTTTAGATGAATTCAAAAAAGAATTTGCAGATAAAGCTGC     167
Staphylococcus-capitis-VCU116_38                   TGGGGTTCTTTAGATGAATTCAAAAAAGAATTTGCAGATAAAGCTGC     167
Staphylococcus-warneri-562-SWAR_20                 TGGGGTTCTTTAGATGAATTCAAAAAAGAATTTGCAGATAAAGCTGC     167
                                                   ***********************************************
```

Suppl. Figure S14. Primers for *S. capitis* orthologous gene sodA are specific and do not recognize other major *Staphylococcus* species (PCR validation).

Quantitative real-time PCR (qPCR) was performed by use of the MyiQ Single-Colour Real-Time Detection System (Bio-Rad laboratories, Hercules, USA) for quantification with Sybr Green and melting curve analysis with *S. capitis* specific primers ScapF/ScapR [6]. Primers were tested on *S. capitis* (1), *S. aureus* (2), *S. epidermidis* (3), and *S. hominis* (4), Marker 200 bp ladder by Stratagene (m). Expected band size 208nt. Sanger sequencing revealed that the band in (1) is indeed 100% identical to the *S. capitis* amplicons as reported in the *in silico* analysis of Suppl. Figure S13.

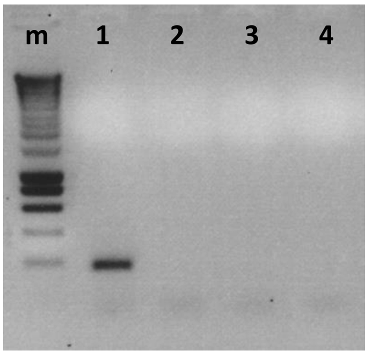

Suppl. Figure S15. qPCR on *S. capitis* validates and replicates findings by SLST.

We performed (**A**) qPCR on *S. capitis* as described in Suppl. Figure S14 and measured its level in our cohort of AD and HC samples (see Supplementary Table S10). Note that 5 out of 28 samples were excluded from analysis because microbiome DNA was no longer available, which also explains why the *p*-value of our original SLST data in (**B**) is slightly different from the earlier reported $p = 0.003$. The *S. capitis* data as measured by qPCR correlates (**C**) very well with the original *S. capitis* data by SLST. The 5 DNA-depleted missing samples are AD1R, AD2L, HC1L, HC1R and HC6R. Statistics by MWU. qPCR levels of *S. capitis* was calculated by 2^-dCt with Broad Range Universal (BRU) 16S rRNA gene primers (forward 5'-3': TCCTACGGGAGGCAGCAGT and reverse 5'-3': GGACTACCAGGGTATCTAATCCTGTT) as the universal bacterial gene-of-reference (see Suppl. Table S16 for the raw qPCR data).

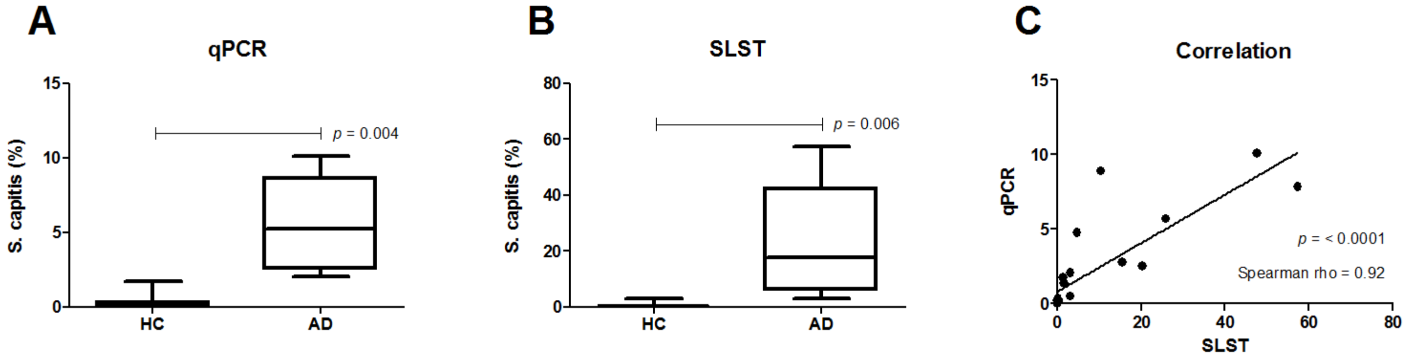

Suppl. Tables S1-S4. Benchmark datasets genome information and statistics.

Genomes used for benchmarking downloaded on: *Bifidobacterium*: 2017 Jan., *Escherichia / Shigella*: 2018 Feb., *P. acnes*: 2016 Jun., *Staphylococcus*: 2016 Apr. Selection of genomes was based on all genomes publicly available at NCBI assembled genomes and NCBI Traces WGS databases [7]. For *Staphylococcus* and *Escherichia / Shigella* a maximum of 200 genomes was selected in order to allow for practically feasible computation times. Therefore, careful effort was undertaken to take into account all available biodiversity based on provided strain annotations. High-quality genomes were first selected (i.e. those assigned as *'reference'* or *'representative'*, thereafter those with closed genomes assemblies or with the lowest number of contigs / scaffolds).

File :
S1-S4 Datasets-for-Benchmarking.xlsx

Suppl. Table S5. 16S and SLST primers used in this study for benchmarking and sequencing.

Method	Region	Primer ID	FW primer (5'-3' sense)	RV primer (5'-3' antisense)	Used for ..	Reference
16S	V1-V2	27Fx338R	AGAGTTTGATCCTGGCTCAG	TGCTGCCTCCCGTAGGAGT	Benchmarking	Baurecht *et al.,* 2018 [8]
16S	V1-V9	8Fx1391R	AGAGTTTGATCCTGGCTCAG	GACGGGCGGTG**W**GT**R**CA	Benchmarking	Kong *et al.,* 2012 [9]
16S	V3-V4	357Fx802RV2	CCTACGGGAGGCAGCAG	TAC**NV**GGGTATCTAA**K**CC	Sequencing	Zeeuwen *et al.,* 2017 [10]
SLST	*30S ribosomal protein S11 (OG #1123)*	not applicable	TGGCACGTAAACAAGTATC	GACGACGTTTTGGTGGAC	Sequencing	Current study (and Smits *et al.,* 2019 [11])

Suppl. Tables S6-S9. Benchmark datasets TaxPhlAn run output reports.

File :
S6-S9 TaxPhlAn-Reference-Runs.xlsx

Suppl. Table S10. Study samples overview Ederveen *et al.*

The samples used in this study are from a recently generated larger cohort from our department by Smits *et al.*, from which we have selected all available baseline samples for which both 16S and SLST sequences were generated [11]. In the study by Smits *et al.,* we studied the effect of coal tar treatment on the skin microbiome; however, the for this study selected samples are from before start of coal tar treatment (hence, baseline) and thus considered the endogenous skin microbiome of these volunteers. "COMB_" samples (from *combination*) are samples with 1:1 pooled amplicons of 16S and SLST of the same volunteer (note that 16S and SLST amplicons were generated from the same DNA sample of each volunteer). The general idea behind the "COMB_" samples is (i) to have biological replicates, and (ii) to test feasibility of combining 16S and SLST amplicons of the same volunteer in one sequencing sample with the same barcodes (i.e. to reduce sequencing costs for application of SLST). * samples codes as reported by Smits *et al.,* [11], these are the same sample identifiers as have been uploaded to ENA. ** sample names are similar for 16S and for SLST, but preceded by "16S_" or "SLST_", respectively (with exception of "COMB_" samples because these contain both 16S and SLST amplicons). AD = atopic dermatitis patient. EASI = Eczema Area and Severity Index. HC = healthy control volunteer.

Sample ID Smits *et al.* *	Sample ID Ederveen *et al.* **	Individual	Sample Site	Arm (L = left, R = right)	Condition	Skin Status	AD Severity (EASI)	Age (years)	Gender (M/F)
AD1_T_0	AD1_L	AD1	inner elbow	L	AD	lesional	13.8	27	M
AD1_V_0	AD1_R	AD1	inner elbow	R	AD	lesional	13.8	27	M
AD2_T_0	AD2_L	AD2	inner elbow	L	AD	lesional	44.4	21	M
AD2_V_0	AD2_R	AD2	inner elbow	R	AD	lesional	44.4	21	M
AD3_T_0	AD3_L	AD3	inner elbow	L	AD	lesional	8.1	27	F
AD3_V_0	AD3_R	AD3	inner elbow	R	AD	lesional	8.1	27	F
AD5_T_0	AD4_L	AD4	inner elbow	L	AD	lesional	25.4	25	F
AD5_V_0	AD4_R	AD4	inner elbow	R	AD	lesional	25.4	25	F
AD7_T_0	AD5_L	AD5	inner elbow	L	AD	lesional	11.6	23	M
AD7_V_0	AD5_R	AD5	inner elbow	R	AD	lesional	11.6	23	M
HC10_T_0	HC1_L	HC1	inner elbow	L	HC	normal	0	30	M
HC10_V_0	HC1_R	HC1	inner elbow	R	HC	normal	0	30	M
HC2_T_0	HC2_L	HC2	inner elbow	L	HC	normal	0	30	F
HC2_V_0	HC2_R	HC2	inner elbow	R	HC	normal	0	30	F
HC3_T_0	HC3_L	HC3	inner elbow	L	HC	normal	0	30	F
HC3_V_0	HC3_R	HC3	inner elbow	R	HC	normal	0	30	F
HC4_T_0	HC4_L	HC4	inner elbow	L	HC	normal	0	35	F
HC4_V_0	HC4_R	HC4	inner elbow	R	HC	normal	0	35	F
HC5_T_0	HC5_L	HC5	inner elbow	L	HC	normal	0	22	F
HC5_V_0	HC5_R	HC5	inner elbow	R	HC	normal	0	22	F
HC6_T_0	HC6_L	HC6	inner elbow	L	HC	normal	0	30	F
HC6_V_0	HC6_R	HC6	inner elbow	R	HC	normal	0	30	F
HC7_T_0	HC7_L	HC7	inner elbow	L	HC	normal	0	51	F
HC7_V_0	HC7_R	HC7	inner elbow	R	HC	normal	0	51	F
HC8_T_0	HC8_L	HC8	inner elbow	L	HC	normal	0	27	M
HC8_V_0	HC8_R	HC8	inner elbow	R	HC	normal	0	27	M
HC9_T_0	HC9_L	HC9	inner elbow	L	HC	normal	0	22	F
HC9_V_0	HC9_R	HC9	inner elbow	R	HC	normal	0	22	F
COMB_AD1_V_0	COMB_AD1_R	AD1	inner elbow	R	AD	lesional	13.8	27	M
COMB_AD2_V_0	COMB_AD2_R	AD2	inner elbow	R	AD	lesional	44.4	21	M
COMB_AD3_T_0	COMB_AD3_L	AD3	inner elbow	L	AD	lesional	8.1	27	F
COMB_AD5_T_0	COMB_AD4_L	AD4	inner elbow	L	AD	lesional	25.4	25	F
COMB_AD7_T_0	COMB_AD5_L	AD5	inner elbow	L	AD	lesional	11.6	23	M

Suppl. Table S11. 16S sequencing read statistics and alpha diversity metrics.

File :
S11 16S-Sequencing-Read-Statistics.xlsx

Suppl. Table S12. 16S analysis of *Staphylococcus* by OTU clustering of full-length 16S, V1-2 and V3-4.

File :
S12 16S-Analysis-Staphylococcus(V1-9)(V1-2)(V3-4).xlsx

Suppl. Table S13. SLST candidates for *Staphylococcus* profiling.

In total, 9 primer sets as listed in this table were initially screened on the lab by PCR with genomic DNA from *S. aureus* strain ATCC 29213 to check for recognition of *Staphylococcus* species. The SLST primer sets marked in green showed positive PCR responses and were further evaluated as reported in Suppl. Fig. S8. The SLST OG #1123 (dark green) is our final SLST target which survived all *in silico* and lab validations, and with which our study was performed. SLST candidates were found by running TaxPhlAn on a set of 200 *Staphylococcus* reference genomes (Table 1, Suppl. Table S9), and their characteristics according to TaxPhlAn default reports and additional *in silico* tests are listed below. The *in silico* PCR were performed on 7085 *Staphylococcus* reference genomes, and on available family Staphylococcaceae ' out-genomes ' and common skin commensals.

Table S13 *part 1 of 2*

#OG	predicted gene product	amplicon length (based on alignment incl. primers)	# of unique sequences (VR), i.e. SLST types	ML-based correlation to phylogeny (rho)	FW len.	RV len.	FW T_m	RV T_m	delta T_m
810	50S ribosomal protein L16	152	46	0.91	19	19	49.0	53.3	4.3
**440**	**Elongation factor G**	**482**	**69**	**0.91**	**20**	**20**	**55.2**	**53.7**	**1.5**
426	Elongation factor G	452	66	0.91	20	20	55.2	49.5	5.7
**1076**	**DNA-directed RNA polymerase subunit beta**	**453**	**80**	**0.89**	**18**	**20**	**46.0**	**51.4**	**5.4**
**1123**	**30S ribosomal protein S11**	**381**	**48**	**0.87**	**19**	**18**	**50.0**	**53.9**	**3.9**
420	Elongation factor T	205	51	0.83	21	19	50.3	53.5	3.2
**1116**	**50S ribosomal protein L16**	**241**	**47**	**0.80**	**20**	**20**	**52.1**	**54.6**	**2.5**
952	50S ribosomal protein L16	203	42	0.78	20	19	50.1	49.3	0.8
435	50S ribosomal protein L1	171	49	0.73	19	19	50.6	52.5	1.9
					primer set predicted characteristics				

Table S13 *part 2 of 2*

#OG	0.2	0.3	0.4	0.5	0.6	0.7	0.8	0.9	FW primer (5'-3' sense)	RV primer (5'-3' antisense)	# Staph. genomes recognized (7085 references)	off-targets based on *in silico* PCR<br><br>* in red distant (undesired) off-targets, in blue off-targets within the family of Staphylococcaceae
810	52	40	32	26	21	9	7	4	GTCAAGGTAAACAAAC**W**CG	ACCGTTACGTCC**W**ACTTCA	7080	
440	80	65	51	36	21	10	6	5	CGTGAACGTGTAGGTCGTTT	ACATCACC**R**TATTGACCACG	7084	
426	76	61	48	34	21	10	6	5	CGTGAACGTGTAGGTCGTTT	TGACGAGAGAATTTACCTTG	7084	Bacillus
1076	80	65	60	51	37	23	17	12	C**W**GGTGAAAAAGTTGAAG	CACCTTGCATACG**R**TAAACT	7063	Macrococcus, Salinicoccus
**1123**	**59**	**51**	**44**	**36**	**27**	**12**	**9**	**4**	**TGGCACGTAAACAAGTATC**	**GACGACGTTTTGGTGGAC**	**7078**	Macrococcus
420	61	49	40	33	26	13	10	5	GC**W**GGTATGATGGATTGTAAA	TACGAGCAACGAAGTC**W**GT	7081	Aliicoccus, Jeotgalicoccus, Nosocomiicoccus
1116	46	29	22	15	13	8	3	1	ATATCGTCGTCAACATCGTC	ACCTTTACCAGCACCCATAC	7082	
952	38	25	18	12	10	7	3	1	CGTGT**W**AAATATCGTCGTCA	GGTGTATG**W**GGGAAGATTT	7082	Bacillus
435	49	43	34	31	27	13	7	3	GTGCTGAAAAAGC**W**GGTAT	CAGGACCCATTGTTGT**W**GT	7076	Bacillus, Macrococcus
	number of SNP per SDI threshold											

Suppl. Table S14-S15. 16S and SLST compositional matrix.

File :
S14-15 16S-and-SLST-Compositional-Tables.xlsx

Suppl. Table S16. Table with raw qPCR data on *S. capitis* in comparison to SLST.

We performed qPCR on *S. capitis* as described in Suppl. Figures S14 and S15 and measured its level in our cohort of AD and HC samples (see Supplementary Table S10).

	16S (Ct)	*S. capitis* (Ct)	delta Ct (dCt)	qPCR fraction to 16S (2^-dCt)	SLST fraction corrected for 16S
**HC2_L**	24.72	32.95	8.23	0.33%	0.16%
**HC3_L**	50.00	50.00	0.00	0.00%	0.00%
**HC4_L**	23.11	32.25	9.14	0.18%	0.13%
**HC5_L**	25.03	34.36	9.33	0.16%	0.29%
**HC6_L**	26.84	50.00	0.00	0.00%	0.01%
**HC7_L**	23.00	31.76	8.76	0.23%	0.14%
**HC8_L**	23.47	29.30	5.84	1.75%	1.40%
**HC9_L**	21.39	37.41	16.02	0.00%	0.01%
**HC2_R**	25.03	31.25	6.22	1.35%	1.60%
**HC3_R**	25.40	34.27	8.87	0.21%	0.07%
**HC4_R**	22.60	31.97	9.37	0.15%	0.15%
**HC5_R**	26.10	34.81	8.72	0.24%	0.21%
**HC7_R**	22.97	32.09	9.12	0.18%	0.26%
**HC8_R**	22.39	30.04	7.65	0.50%	3.14%
**HC9_R**	23.33	35.49	12.16	0.02%	0.04%
**AD1_L**	20.29	24.67	4.39	4.78%	4.64%
**AD3_L**	22.45	25.94	3.49	8.90%	10.43%
**AD4_L**	23.04	28.36	5.32	2.50%	20.24%
**AD5_L**	19.05	22.36	3.31	10.10%	47.71%
**AD2_R**	20.44	26.04	5.60	2.07%	3.12%
**AD3_R**	20.77	24.90	4.13	5.71%	25.84%
**AD4_R**	24.00	29.17	5.17	2.78%	15.55%
**AD5_R**	17.74	21.42	3.67	7.84%	57.33%

References of Supplementary Data

1. Rice, P., I. Longden, and A. Bleasby, *EMBOSS: the European Molecular Biology Open Software Suite.* Trends Genet, 2000. **16**(6): p. 276-7.
2. Price, M.N., P.S. Dehal, and A.P. Arkin, *FastTree: computing large minimum evolution trees with profiles instead of a distance matrix.* Mol Biol Evol, 2009. **26**(7): p. 1641-50.
3. Letunic, I. and P. Bork, *Interactive tree of life (iTOL) v3: an online tool for the display and annotation of phylogenetic and other trees.* Nucleic Acids Res, 2016. **44**(W1): p. W242-5.
4. Edgar, R.C., *MUSCLE: multiple sequence alignment with high accuracy and high throughput.* Nucleic Acids Res, 2004. **32**(5): p. 1792-7.
5. Walters, W.A., et al., *PrimerProspector: de novo design and taxonomic analysis of barcoded polymerase chain reaction primers.* Bioinformatics, 2011. **27**(8): p. 1159-61.
6. Iwase, T., et al., *Development of a real-time PCR assay for the detection and identification of Staphylococcus capitis, Staphylococcus haemolyticus and Staphylococcus warneri.* J Med Microbiol, 2007. **56**(Pt 10): p. 1346-9.
7. Kitts, P.A., et al., *Assembly: a resource for assembled genomes at NCBI.* Nucleic Acids Res, 2016. **44**(D1): p. D73-80.
8. Baurecht, H., et al., *Epidermal lipid composition, barrier integrity, and eczematous inflammation are associated with skin microbiome configuration.* Journal of Allergy and Clinical Immunology, 2018. **141**(5): p. 1668-1676.e16.
9. Kong, H.H., et al., *Temporal shifts in the skin microbiome associated with disease flares and treatment in children with atopic dermatitis.* Genome Res, 2012. **22**(5): p. 850-9.
10. Zeeuwen, P.L.J.M., et al., *Reply to Meisel et al.* Journal of Investigative Dermatology, 2017. **137**(4): p. 961-962.
11. Smits, J.P.H., et al., *TARgeting the cutaneous microbiota in atopic dermatitis by coal tar via AHR-dependent induction of antimicrobial peptides.* J Invest Dermatol, 2019. **[Epub Ahead of Print] doi: 10.1016/j.jid.2019.06.142**.

Supplementary Figure Files

A generic workflow for Single Locus Sequence Typing (SLST) design and subspecies characterization of microbiota.

Thomas H.A. Ederveen 1-3 #, Jos P.H. Smits 2, Karima Hajo 1, Saskia van Schalkwijk 3, Tessa A. Kouwenhoven 2, Sabina Lukovac 3, Michiel Wels 1,3, Ellen H. van den Bogaard 2, Joost Schalkwijk 2, Jos Boekhorst 1,3, Patrick L.J.M. Zeeuwen 2 and Sacha A.F.T. van Hijum 1,3

1. Center for Molecular and Biomolecular Informatics (CMBI), Radboud Institute for Molecular Life Sciences (RIMLS), Radboud University Medical Center (Radboudumc), Nijmegen, The Netherlands.

2. Department of Dermatology, RIMLS, Radboudumc, Nijmegen, The Netherlands.

3. NIZO, Ede, The Netherlands.

# Corresponding author: Thomas H.A. Ederveen, Center for Molecular and Biomolecular Informatics (CMBI), Radboud Institute for Molecular Life Sciences (RIMLS), Radboud University Medical Center (Radboudumc), Geert Grooteplein Zuid 28 (route 260), 6525 GA, Nijmegen, The Netherlands. E: Tom.Ederveen@radboudumc.nl, T: 0031-243619390

This combined PDF contains the following files :

Files for Supplementary Figures :
S3 Bifidobacterium-SLST-ID-630.24-34.21-21.iTOL.pdf
S4 Escherichia-Shigella-SLST-ID-1978.3-8.32-32.iTOL.pdf
S5 Propionibacterium-SLST-ID-1414.3-7.9-9.iTOL.pdf
S6 Staphylococus-SLST-ID-1238.2-13.3-3.iTOL.pdf
S7 SLST-Staphylococcus-OG1123.pdf

N.B. Other supplementary Figures not mentioned here can be found in the supplementary Data document.

S3 Bifidobacterium-SLST-ID-630.24-34.21-21.iTOL.pdf

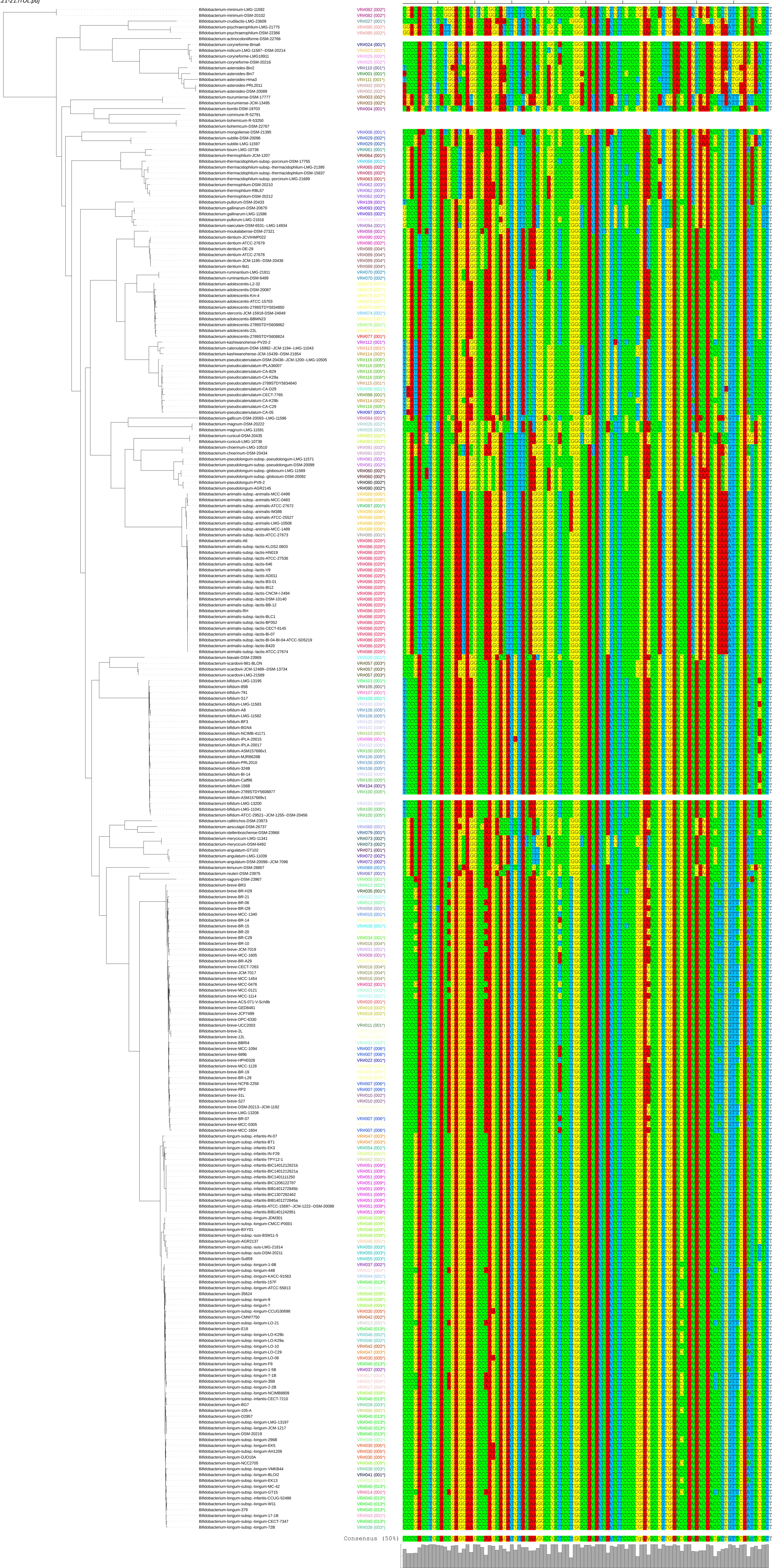

S4 Escherichia-Shigella-SLST-ID-1978.3-8.32-32.iTOL.pdf

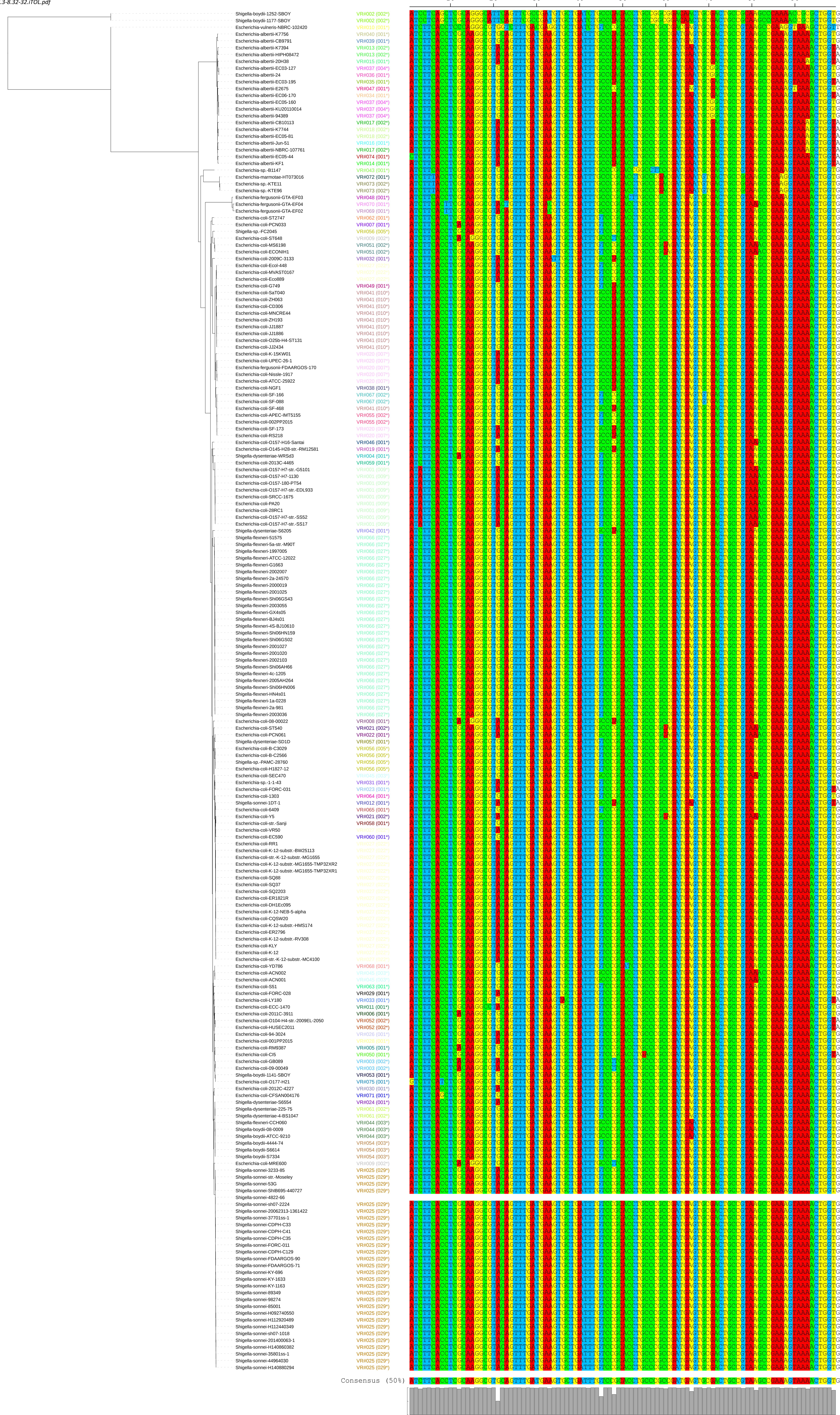

S5 Propionibacterium-SLST-ID-1414.3-7.9-9.iTOL.pdf

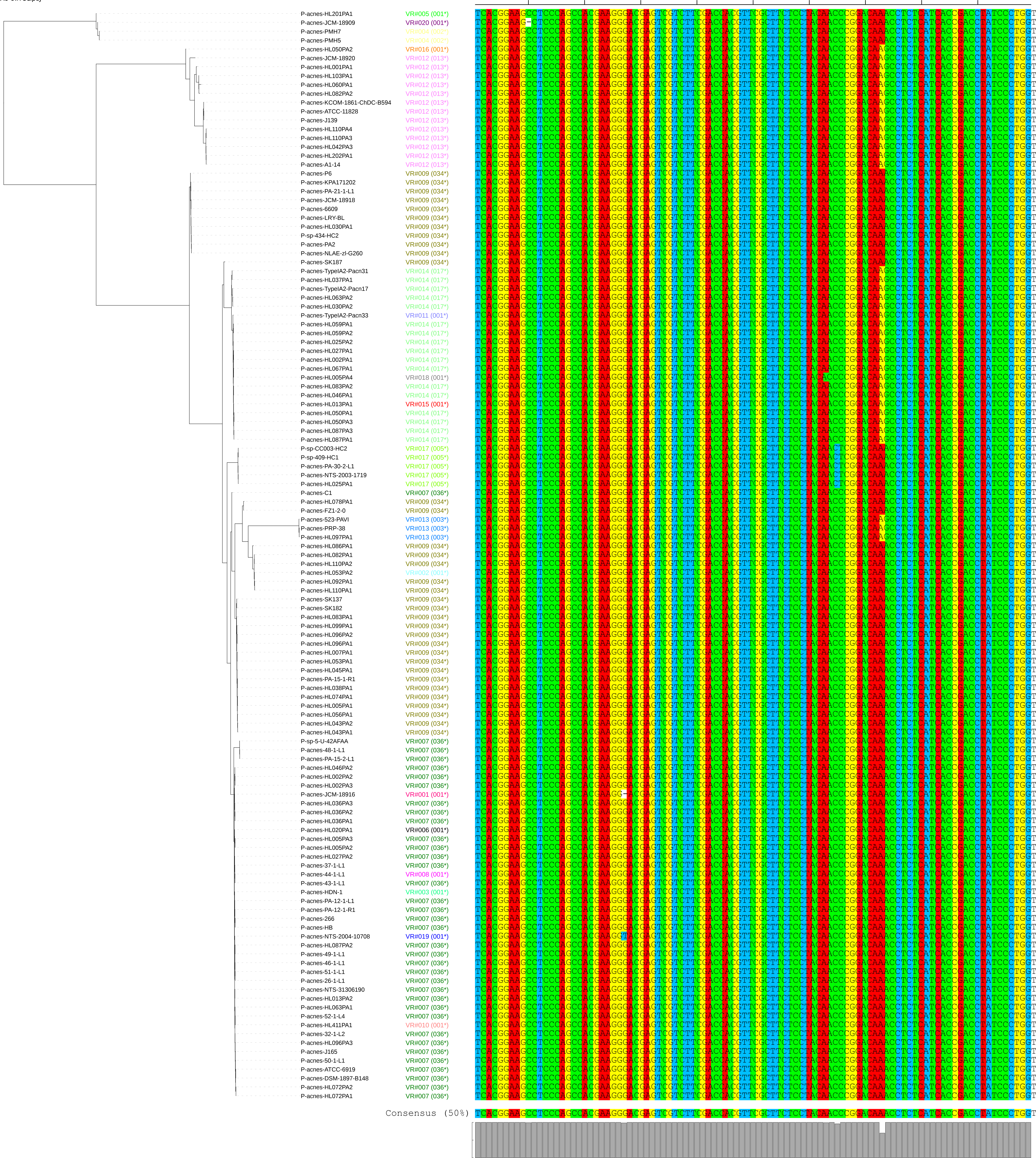

S6 Staphylococus-SLST-ID-1238.2-13.3-3.iTOL.pdf

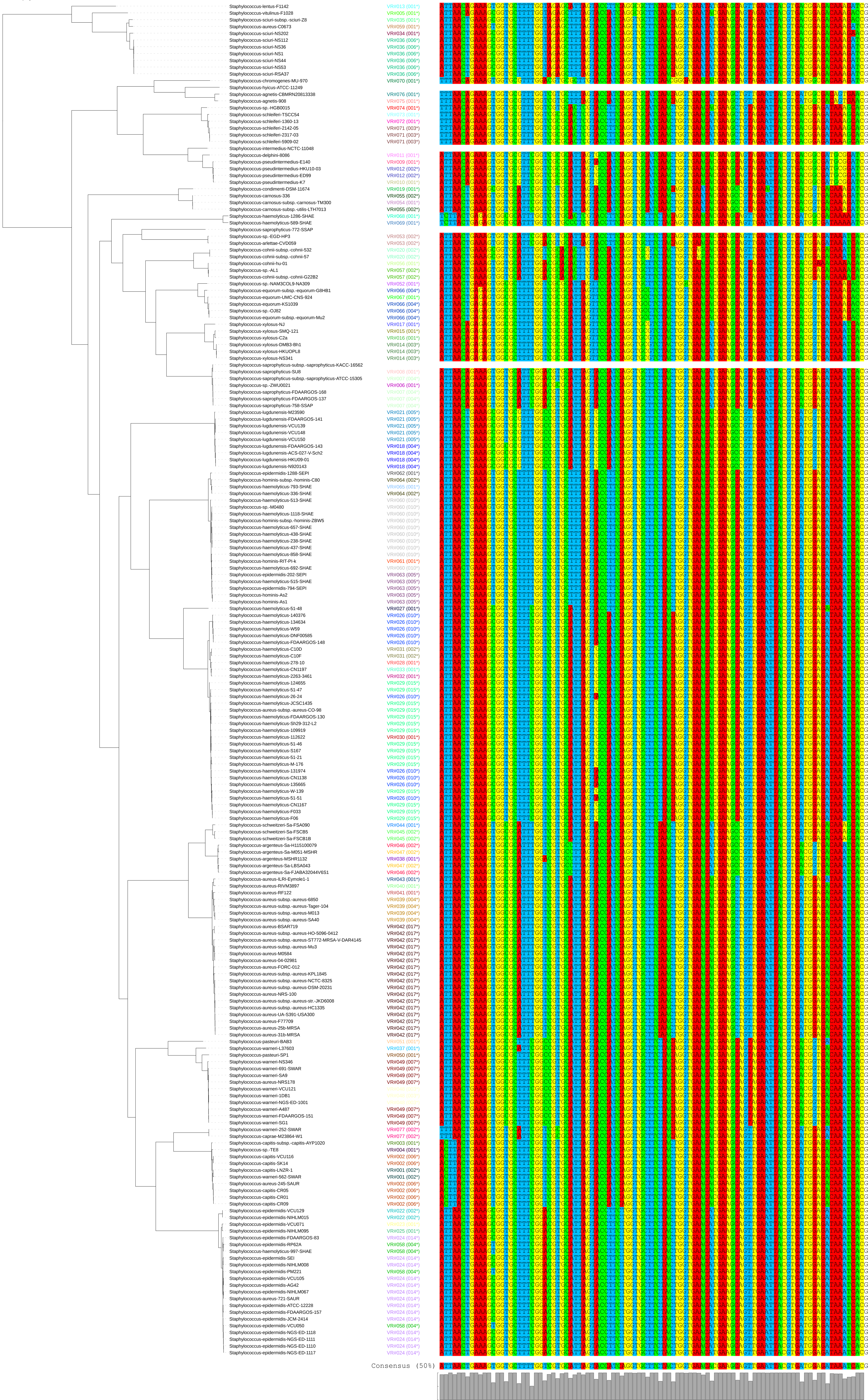

S7 SLST-Staphylococcus-OG1123.pdf

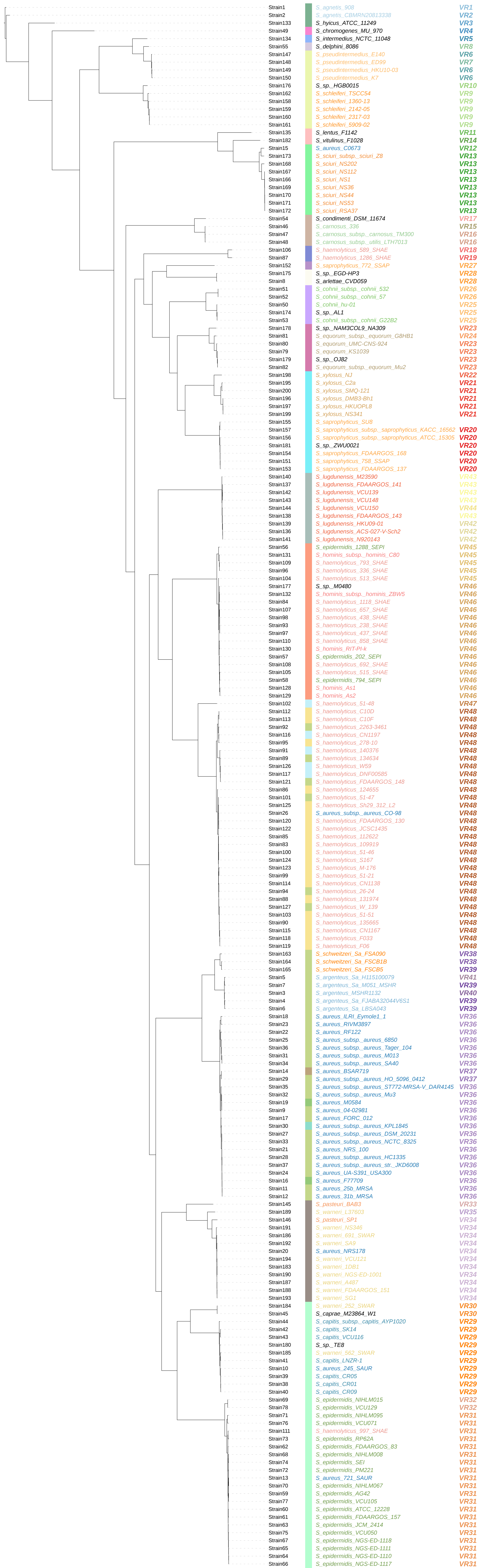

Supplement: Supplementary file 1 — Supplementary Information [file 41598_2019_56065_MOESM1_ESM.pdf]
